# RESC14 and RESC8 cooperate to mediate RESC function and dynamics during trypanosome RNA editing

**DOI:** 10.1093/nar/gkae561

**Published:** 2024-07-05

**Authors:** Katherine Wackowski, Xiaoyu Zhu, Shichen Shen, Ming Zhang, Jun Qu, Laurie K Read

**Affiliations:** Department of Microbiology and Immunology, Jacobs School of Medicine and Biomedical Sciences, Buffalo, NY 14203, USA; Department of Pharmaceutical Sciences, University at Buffalo, Buffalo, NY 14214, USA and NYS Center of Excellence in Bioinformatics and Life Sciences, University at Buffalo, Buffalo, NY 14203, USA; Department of Pharmaceutical Sciences, University at Buffalo, Buffalo, NY 14214, USA and NYS Center of Excellence in Bioinformatics and Life Sciences, University at Buffalo, Buffalo, NY 14203, USA; Department of Pharmaceutical Sciences, University at Buffalo, Buffalo, NY 14214, USA and NYS Center of Excellence in Bioinformatics and Life Sciences, University at Buffalo, Buffalo, NY 14203, USA; Department of Pharmaceutical Sciences, University at Buffalo, Buffalo, NY 14214, USA and NYS Center of Excellence in Bioinformatics and Life Sciences, University at Buffalo, Buffalo, NY 14203, USA; Department of Microbiology and Immunology, Jacobs School of Medicine and Biomedical Sciences, Buffalo, NY 14203, USA

## Abstract

Mitochondrial transcripts in *Trypanosoma brucei* require extensive uridine insertion/deletion RNA editing to generate translatable open reading frames. The RNA editing substrate binding complex (RESC) serves as the scaffold that coordinates the protein–protein and protein–RNA interactions during editing. RESC broadly contains two modules termed the guide RNA binding complex (GRBC) and the RNA editing mediator complex (REMC), as well as organizer proteins. How the protein and RNA components of RESC dynamically interact to facilitate editing is not well understood. Here, we examine the roles of organizer proteins, RESC8 and RESC14, in facilitating RESC dynamics. High-throughput sequencing of editing intermediates reveals an overlapping RESC8 and RESC14 function during editing progression across multiple transcripts. Blue native PAGE analysis demonstrates that RESC14 is essential for incorporation of RESC8 into a large RNA-containing complex, while RESC8 is important in recruiting a smaller ribonucleoprotein complex (RNP) to this large complex. Proximity labeling shows that RESC14 is important for stable RESC protein–protein interactions, as well as RESC–RECC associations. Together, our data support a model in which RESC14 is necessary for assembly of editing competent RESC through recruitment of an RNP containing RESC8, GRBC and gRNA to REMC and mRNA.

## Graphical Abstract

**Graphical Abstract:**
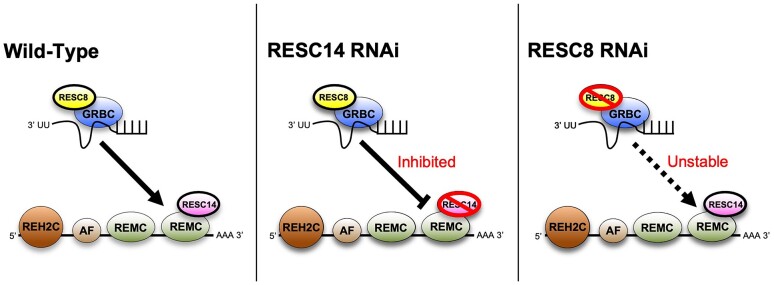

## Introduction

Kinetoplastids are a group of flagellated protozoans that are distinguished by the presence of a unique mitochondrial DNA structure called the kinetoplast. In *Trypanosoma brucei*, the causative agent of Human African Trypanosomiasis, the kinetoplast is comprised of a concatenated network of approximately 50 maxicircles (∼23 kb) and thousands of minicircles (∼1 kb) (1). Maxicircles contain 18 protein-coding genes that encode for components of the mitochondrial respiratory chain and mitochondrial ribosome, as well as two mitochondrial rRNAs. Twelve of these 18 mRNAs require extensive posttranscriptional modification, during which uridines (Us) are inserted and less frequently deleted by RNA editing to generate translatable open reading frames (2,3). This process is essential to the survival of both the procyclic form (PF) and mammalian bloodstream form of *T. brucei* (4,5). *Trans*-acting guide RNAs (gRNAs) are primarily encoded in minicircles and direct the precise location of U insertion and deletion through gRNA–mRNA base pairing interactions (6–8). Nine out of the 12 edited mRNAs are called ‘pan-edited’ and require dozens of gRNAs to edit throughout the entire length of the mRNAs. Three transcripts are considered ‘moderately edited’ and require only one or two gRNAs to edit a small region (2).

The process of U insertion/deletion (U-indel) RNA editing requires the 3′ to 5′ sequential use of gRNAs to fully edit a pan-edited transcript. Editing is initiated when the 5′ anchor region of the first gRNA hybridizes to the complementary 3′ end of a pre-edited mRNA. The central region of the gRNA then serves as the template that directs U insertion and deletion, where the gRNA and mRNA interact through Watson–Crick and wobble G–U base pairing. Editing is completed through the length of the initiating gRNA when the gRNA–mRNA are completely complementary. The gRNA–mRNA duplex is then separated by an unknown mechanism, allowing the subsequent gRNA to anchor (9,10). Although editing proceeds in the general 3′ to 5′ direction along an mRNA, many partially edited mRNAs (mRNAs in the process of being edited) contain mis-edited sequences at the leading 5′ edge of editing called junctions. Junctions are edited sequences that do not match the canonically fully edited or pre-edited sequences and are thought to represent both areas of active editing and dead-end products (10–13).

The RNA editing holoenzyme contains three dynamically acting, multiprotein complexes that are responsible for carrying out U-indel RNA editing: the RNA Editing Catalytic Complexes (RECCs), the RNA Editing Substrate Binding Complex (RESC), and the RNA Editing Helicase 2 Complex (REH2C) (2). The RECCs contain 19 stably-bound proteins that are responsible for the enzymatic reactions of RNA editing, including endonuclease cleavage, addition/deletion of uridines, and RNA ligation. Despite containing the enzymes for catalysis, the RECCs contain little RNA and lack processivity *in vivo* (14,15). RESC and REH2C stably bind mRNA and interact transiently with the RECCs via RNA interactions (2,15–17). REH2C includes the DEAH/RHA type helicase, KREH2, as well as two associated cofactors and affects total editing and accuracy of editing on transcripts bound to RESC (18,19). Another RNA helicase, KREH1, is one of many editing accessory factors. KREH1 promotes initiator gRNA utilization on numerous mRNAs (20). Current models envision RESC as a scaffold that coordinates interactions between the gRNAs, mRNAs, RECCs and REH2C, as well as with other RNA processing complexes and accessory factors; however, the mechanisms by which RESC coordinates these interactions are not well understood (2).

RESC consists of ∼20 proteins organized into dynamically interacting modules: the Guide RNA Binding Complex (GRBC; a.k.a. RESC-A (21) and MRB1 core (22)) and the RNA Editing Mediator Complex (REMC) (15,22). In addition to these subcomplexes, at least three proteins act as RESC organizers (RESC8, RESC10 and RESC14) and are needed for complete GRBC-REMC protein interactions and proper gRNA/mRNA interactions with RESC (23–25). When either RESC8 or RESC14 are knocked down by RNAi, GRBC and REMC disassociate, but the protein components of each module stay together (24,25). The phenotype of RESC10 RNAi cells is unique among RESC organizers in that, in addition to GRBC-REMC dissociation, the integrity of GRBC is also lost as the RESC5 and RESC6 interaction is compromised (23).

Supporting the idea that RESC itself is a dynamic complex, a recent cryogenic electron microscopy (cryo-EM) study defined different RESC complexes whose composition indicates that RESC protein and RNA rearrangements must occur during editing (21). The study showed gRNA-bound GRBC (comprised of RESC1-6) must interact with mRNA-bound RESC organizers and REMC (comprising RESC9, RESC11–13, and potentially RESC7), forming the editing-competent substrate (RESC5-14, a.k.a. RESC-B) that is presumably capable of productive interaction with the RECCs (21). The composition of the cryo-EM defined complexes necessitates an RNA/protein remodeling step that includes expulsion of RESC1-4 prior to the start of active editing. There must also be a complex disassembly during gRNA removal, possibly resulting in the cryo-EM-defined RESC-C subcomplex comprised of RESC5-8, RESC10 and RESC14. How this protein/RNA remodeling occurs and the precise roles of RESC organizers during the progression of the editing are not understood. The cryo-EM data show that RESC8, RESC10 and RESC14 are positioned in-between GRBC and REMC, supporting the findings that these organizers facilitate proper interactions between these subcomplexes (23–25). RESC10 is also positioned between GRBC components RESC5 and RESC6 post-rearrangement, supporting its role in stabilizing their interaction (23). The focus of this study is to better understand the functions of RESC organizers, RESC8 and RESC14, and their roles in modulating RESC protein/RNA dynamics. Our data is consistent with a model in which mRNA-bound RESC14 signals RESC8 to assemble GRBC and a gRNA with mRNA-bound REMC, forming the editing competent version of RESC.

## Materials and methods

### Generation of *T. brucei* cell lines

All cell lines used in this study were derived from PF *T. brucei* 29–13 cells. Cells were grown at 27°C in standard media supplemented with 10% fetal bovine serum as previously described (26,27). To generate endogenous, C-terminal Myc-His-TAP (MHT)-tagged RESC14 cells, a PCR product produced through long primer PCR of pPOTv4 containing the MHT tag, and protein-specific primers (Supplemental Table S1) was transfected into 29–13 cells (28). Transformants were selected using 1 μg/ml puromycin, and clones were obtained using limiting dilution. To generate cell lines harboring both RESC14 RNAi and C-terminal MHT-tagged RESC8, RESC11A, RESC12A, RESC13, KREH2, KREH1 or KREPB5, the pPOTv4-MHT-puromycin cassette was amplified using gene-specific primers (Supplemental Table S1) and transfected into previously generated RESC14 RNAi cells (25,28). Transformants were selected using 1 μg/ml puromycin and 2.5 μg/ml phleomycin, and clones were obtained using limiting dilution. To generate a cell line containing Protein A-TEV-Protein C (PTP)-tagged RESC2 in the RESC14 RNAi background, the RESC2 open reading frame (ORF) was PCR-amplified with the addition of 5′ ApaI and 3′ BamHI restriction sites (Supplemental Table S1). The ApaI- and BamHI-digested PCR product was then cloned into a similarly digested pC-PTP plasmid (29). The plasmid was linearized with XhoI and transfected into previously generated RESC14 RNAi cells (25) and selected with 1 μg/ml puromycin. RESC6-PTP in the RESC14 RNAi background were previously generated (25). For all cell lines harboring RESC14 RNAi, RNAi was induced by adding 4 μg/ml doxycycline to cell cultures for three days.

A cell line containing both MHT-tagged RESC14 and RESC8 RNAi was also generated in this study using the same long primer PCR method mentioned above, and previously generated RESC8 RNAi cells (24). RESC6-PTP in the RESC8 RNAi background was previously generated (24). Knockdown of RESC8 was induced using 4 μg/ml doxycycline for 2 days. The RESC13 RNAi cell line used in this study was previously generated (22). RESC13 RNAi was induced using 4 μg/ml doxycycline for two days.

To generate TurboID-tagged RESC6 and RESC8, the ORF of TurboID with an HA tag and puromycin resistance cassette were PCR-amplified using the plasmid pJB1231, a derivative of pXS6 (30), as a template with our long primer PCR method. This PCR product was transfected into the RESC14 RNAi cells (25). Transformants were selected using 1 μg/ml puromycin and 2.5 μg/ml phleomycin, and clones were obtained using limiting dilution. All primers used in this study are listed in Supplemental Table S1, and all cell lines used in this study are listed in Supplemental Table S2.

### High-throughput sequencing and bioinformatic analysis

PF *T. brucei* RESC8 RNAi cells were grown in the presence or absence of 4 μg/ml doxycycline for two days (24). RNA was isolated using Trizol (Invitrogen) and phenol:chloroform extraction and ethanol precipitation, followed by DNase treatment with a DNA-free DNase Kit (Ambion). Two biological replicates were performed, and qRT-PCR was used to validate the level of RESC8 knockdown (∼30% remaining). The DNase-treated RNA was converted to cDNA with the Superscript III Reverse Transcription Kit (Invitrogen) and gene-specific primers for the 3′ region of cytochrome oxidase subunit III (COIII) and ATPase subunit 6 (A6) mRNAs were used for amplification of these cDNAs as previously described (31). Due to sequencing discrepancies at the 5′ end of the amplified COIII section, we have designated the transcript end five editing sites (ES) 3′ of the true 5′ end. This means that ‘fully edited’ COIII refers to where the canonical edited sequence matches up to ES 109 as previously described (32). The gene-specific cDNAs were PCR-amplified in the linear range to ensure the relative abundance of unique fragments was maintained. Library preparation and paired-end Illumina MiSeq sequencing was then performed as previously described (32). Two replicates of induced RESC8 RNAi were compared to two replicates of uninduced RESC8 RNAi samples combined with five PF 29–13 cells from another study (31). Sample preparation and controls were previously described for COIII and A6 mRNAs from RESC13 and RESC14 RNAi lines (32), and for Ribosomal Protein S12 (RPS12) mRNA from RESC8 (24), RESC13 (11) and RESC14 RNAi lines (25).

The number of standard reads (sequences with no non-T mismatches) and nonstandard reads (sequences with non-T mismatches) are listed in Supplemental Table S3. To compare relative abundances of specific sequences between samples, reads in each sample were normalized to 100 000 counts (33). The nonstandard reads were excluded from the analysis and the normalized reads were aligned to the published pre-edited and fully edited A6 and COIII mRNA sequences (31) using the Trypanosome Editing Alignment Tool (TREAT), which allows us to analyze large populations of mitochondrial mRNAs at single nucleotide resolution. The new sequencing data for A6 and COIII, RESC8 RNAi samples has been deposited in the Sequence Read Archive under accession number PRJNA986128. Previously published sequencing data is under PRJNA862535 for the A6 and COIII, RESC13 and RESC14 RNAi samples, PRJNA597932 for the A6 and COIII PF 29–13 samples, PRJNA431762 for the RPS12 RESC8 RNAi samples, PRJNA390283 for the RPS12 RESC14 RNAi samples, and PRJNA363102 for the RPS12 RESC13 RNAi and RPS12 control samples.

Determining RESC8 Exacerbated Pause Sites (EPS) and calculating the significance of EPS overlap between the RNAi of different proteins on A6 and COIII mRNAs were both previously described (11) (Supplemental Tables S4 and S5). For junction length analysis, we determined the percent of sequences generated at each editing stop site for A6, COIII and RPS12 mRNAs with a junction length of 0, 1–10, 11–20 and >20 ES long. The average number of sequences for the uninduced and induced RESC8, RESC13 and RESC14 RNAi samples was calculated and plotted in RStudio. Student's *t*-tests in RStudio were performed to calculate editing stop sites where sequences with junction lengths of 0 or >20 were significantly different in the induced RNAi samples compared to the uninduced controls.

### Isolation of crude mitochondria and blue native PAGE analysis

Crude mitochondria were isolated using hypotonic lysis as described previously (34). Briefly, 2.5 × 10^8^ cells were harvested and washed in NET buffer (150 mM NaCl, 100 mM EDTA, 10 mM Tris pH 8.0). Pellets were resuspended in DTE buffer (1 mM Tris pH 8.0, 1 mM EDTA) and homogenized by passing the sample through a 25 G needle three times. A 60% sucrose solution was added immediately after to a final concentration of 250 mM and lysates were cleared by spinning at 15 000×g for 10 min at 4°C. Pellets were resuspended in STM buffer (250 mM sucrose, 20 mM Tris pH 8.0, 2 mM MgCl_2_) and extra MgCl_2_ and CaCl_2_ were added to final concentrations of 3 and 0.3 mM, respectively. Samples were then DNase-treated for 1 h using a final concentration of 14 μg/ml DNase I (Sigma). An equal volume of STE buffer (250 mM sucrose, 20 mM Tris pH 8.0, 10 mM EDTA) was added to the sample to stop DNase I digestion, and samples were centrifuged at 15 000×g for 10 min at 4°C. The final pellet was washed with STE buffer, split equally into four tubes (∼6.3 × 10^7^ cell equivalents each), and stored at –80°C until use.

The crude mitochondrial pellets were analyzed by blue native PAGE, as similarly described (35). Pellets (∼6.3 × 10^7^ cell equivalents) were resuspended in Solubilization Buffer (50 mM NaCl, 50 mM Bis–Tris pH 7.0, 2 mM ϵ-aminocaproic acid (ACA), 1 mM EDTA, cOmplete EDTA-free protease inhibitor cocktail (Sigma)) with 1% digitonin and 0.32 U SUPERase-In™ RNase inhibitor (Invitrogen). For RNase-treated samples, a nuclease cocktail containing 0.05 μg RNase A, 2U RNase T1 (Ambion) and 0.02U RNase H (Invitrogen) was added without RNase inhibitor. Samples were incubated on ice for 1 hr and centrifuged at 16 000×g for 20 min at 4°C. Portions of the supernatants were mixed with glycerol to a final concentration of 5% w/v and native loading dye (500 mM ACA, 5% (w/v) Coomassie Brilliant Blue G250) and run on 3–12% Bis–Tris NativePAGE™ gels (Invitrogen). After electrophoresis (30 min, 160 V, 4°C; 2.5 h, 120 V, 4°C; 30 min, 160 V, 4°C), gels were incubated in a 2.5% SDS denaturing buffer for 15 min, and proteins were transferred to a nitrocellulose membrane. The membrane was probed with peroxidase anti-peroxidase (PAP), which recognizes the Protein A region of the MHT or PTP tag.

### TurboID proximity labeling

TurboID-tagged RESC6 and RESC8 in the RESC14 RNAi background and parental 29–13 cells were harvested at 3 × 10^9^ cells. RESC14 RNAi was induced by adding 4 μg/ml doxycycline to cell cultures for three days. Prior to harvest, cells were incubated with 100 μM of biotin for 20 min. Hypotonic mitochondrial isolation and streptavidin pulldown were performed as previously described (36) with some modifications. Briefly, mitochondrial-enriched pellets were resuspended in 500 μl of Boiling Buffer (1% SDS, 1 mM EDTA, 50 mM Tris pH 7.5) and placed at 80°C for 10 min. Samples were spun at 16 000×g for 10 min at room temperature, and supernatants were mixed with 700 μl of Incubation Buffer (150 mM NaCl, 5 mM EDTA, 1% Triton X-100, 50 mM Tris pH 7.5). 0.5 mg of washed MyOne™ Streptavidin Dynabeads C1 (Invitrogen) were added to samples, rocked at room temperature for 1 h, and moved to a rocker at 4°C overnight. The Dynabeads were stringently washed as previously described (37). Briefly, samples were washed twice with Wash Buffer 1 (2% SDS), once with Wash Buffer 2 (0.1% deoxycholate, 1% Triton X-100, 500 mM NaCl, 1 mM EDTA, 50 mM HEPES pH 7.5), once with Wash Buffer 3 (250 mM LiCl, 0.5% NP-40, 0.5% deoxycholate, 1 mM EDTA) and twice with Wash Buffer 4 (50 mM Tris pH 7.5, 50 mM NaCl). Dynabeads were stored at –80°C until further mass spectrometry analysis. Three replicates of each condition were performed.

### Interactome analysis of TurboID samples

#### On-beads protein digestion

A surfactant-aided precipitation/on-pellet digestion protocol was adopted using our previously published method with slight modification (38). 5% SDS was spiked into each sample to a final concentration of 0.5%. Proteins on the beads were sequentially reduced by 10 mM dithiothreitol (DTT) at 56°C for 30 min and alkylated by 25 mM iodoacetamide (IAM) at 37°C in darkness for 30 min. Both steps were performed in a thermomixer (Eppendorf) with rigorous shaking. The beads with proteins were then precipitated by addition of 6 volumes of chilled acetone with vortexing, and the mixture was incubated at –20°C for 3 h. Samples were then centrifuged at 20 000×g at 4°C for 30 min, and supernatant was removed. The bead pellet was gently rinsed by adding 500 μl methanol, centrifuged again at 20 000×g at 4°C for 30 min, methanol was carefully removed, and air-dried for 1 min. The bead pellet was re-suspended in 46 μl 50 mM pH 8.4 Tris-formic acid (FA). A total volume of 4 μl trypsin (Sigma Aldrich) re-constituted in 50 mM pH 8.4 Tris-FA to a final concentration of 0.25 μg/μl was added for 6 h tryptic digestion at 37°C with constant shaking in a thermomixer. Digestion was terminated by addition of 0.5 μl FA, and samples were centrifuged at 20 000×g at 4°C for 30 min. Supernatant was carefully transferred to liquid chromatography (LC) vials for analysis.

#### LC–MS analysis

The LC–MS system consists of a Dionex Ultimate 3000 nano LC system, a Dinoex Ultimate 3000 micro LC system with a WPS-3000 autosampler, and an Orbitrap Fusion Lumos mass spectrometer. A large-inner diameter (i.d.) trapping column (300 μm i.d. × 5 mm) was implemented before the separation column (75 μm i.d. × 65 cm, packed with 2.5 μm Xselect CSH C18 material) for high-capacity sample loading, cleanup and delivery. For each sample, 10 μl derived peptides was injected for LC–MS analysis. Mobile phase A and B were 0.1% FA in 2% ACN and 0.1% FA in 88% ACN. The 90-min LC gradient profile was: 4% B for 3 min, 4–9% B for 2 min, 9–38% B for 70 min, 90% B for 5 min, and then equilibrated to 4% B for 10 min. The mass spectrometer was operated under data-dependent acquisition (DDA) mode with a maximal duty cycle of 3 s. MS1 spectra was acquired by Orbitrap (OT) under 120k resolution for ions within the m/z range of 400–1500. Automatic Gain Control (AGC) and maximal injection time was set at 175% and 50 ms, and dynamic exclusion was set at 60 s, ±10 ppm. Precursor ions were isolated by quadrupole using a *m*/*z* window of 1.6 Th and were fragmented by high-energy collision dissociation (HCD). MS2 spectra of a precursor ion fragmented were acquired by Ion Trap (IT), which was operated under Rapid scan rate with a Standard AGC target and a maximal injection time of 150 ms. Detailed LC–MS settings and relevant information can be found in a previous publication by Shen *et al.* (39).

#### Data processing

LC–MS files were searched against *Trypanosoma brucei brucei* TREU927 TriTryp database containing 10 642 gene entries (ver February 2021) using Sequest HT embedded in Proteome Discoverer 1.4 (Thermo Fisher Scientific). Target-decoy approach using a concatenated forward and reverse protein sequence database was applied for FDR estimation and control. Searching parameters include: (i) precursor ion mass tolerance: 20 ppm; (ii) product ion mass tolerance: 0.8 Da; (iii) maximal missed cleavages per peptide: 2; (iv) fixed modifications: cysteine (C) carbamidomethylation; (v) dynamic modifications: methionine (M) oxidation, peptide N-terminal acetylation. Search result merging, protein inference/grouping, and FDR control were performed in Scaffold 5 (Proteome Software, Inc.). For identification, global protein/peptide FDR was set to 1.0% and at least two unique peptides were required for each protein. For quantification, protein abundance was determined by total spectrum counts and total MS2 ion intensities. Results were exported and manually curated in Microsoft Excel.

### Immunoprecipitation experiments

Cell lines harboring either endogenously tagged RESC14-MHT or RESC8-MHT were harvested at 9.6 × 10^9^–1.4 × 10^10^ cells, then washed in 1× PBS. Immunoprecipitation was performed as described previously (25). Briefly, cell pellets were lysed in N150 buffer (50 mM Tris pH 8.0, 150 mM NaCl, 0.1% (v/v) NP-40, 5 mM β-ME, cOmplete EDTA-free protease inhibitor cocktail (Sigma)) supplemented with 1% (v/v) Triton X-100 and 1 mM CaCl_2_. Lysate was divided into two fractions: one was incubated with 200 U SUPERase-In™ RNAse inhibitor (Invitrogen) and 0.5 μg/ml DNase I, while the other was incubated with DNase I and a nuclease cocktail containing 60 μg RNase A, 2500 U RNase T1 (Ambion), 28 U RNase H (Invitrogen), and 2040 U micrococcal nuclease for 1 h on ice. The two samples were then incubated with IgG Sepharose 6 Fast Flow beads (GE Healthcare) for 2 h at 4°C. Beads were washed with N150 buffer and then incubated in TEV Cleavage Buffer (10 mM Tris pH 8.0, 150 mM NaCl, 0.1% (v/v) NP-40, 0.5 mM EDTA, 1 mM DTT) with 100 U AcTEV™ Protease (Invitrogen) at 4°C overnight. TEV elutions were subjected to western blot analysis to detect the target protein (either RESC14 or RESC8) using anti-Myc (Invitrogen) and interacting proteins using antibodies specific for RESC6 (40), RESC8 (24), RESC10 (23) and RESC14 (25).

## Results

### RESC14 has an overlapping function with RESC8 during editing progression on large pan-edited transcripts

**Figure 1.**
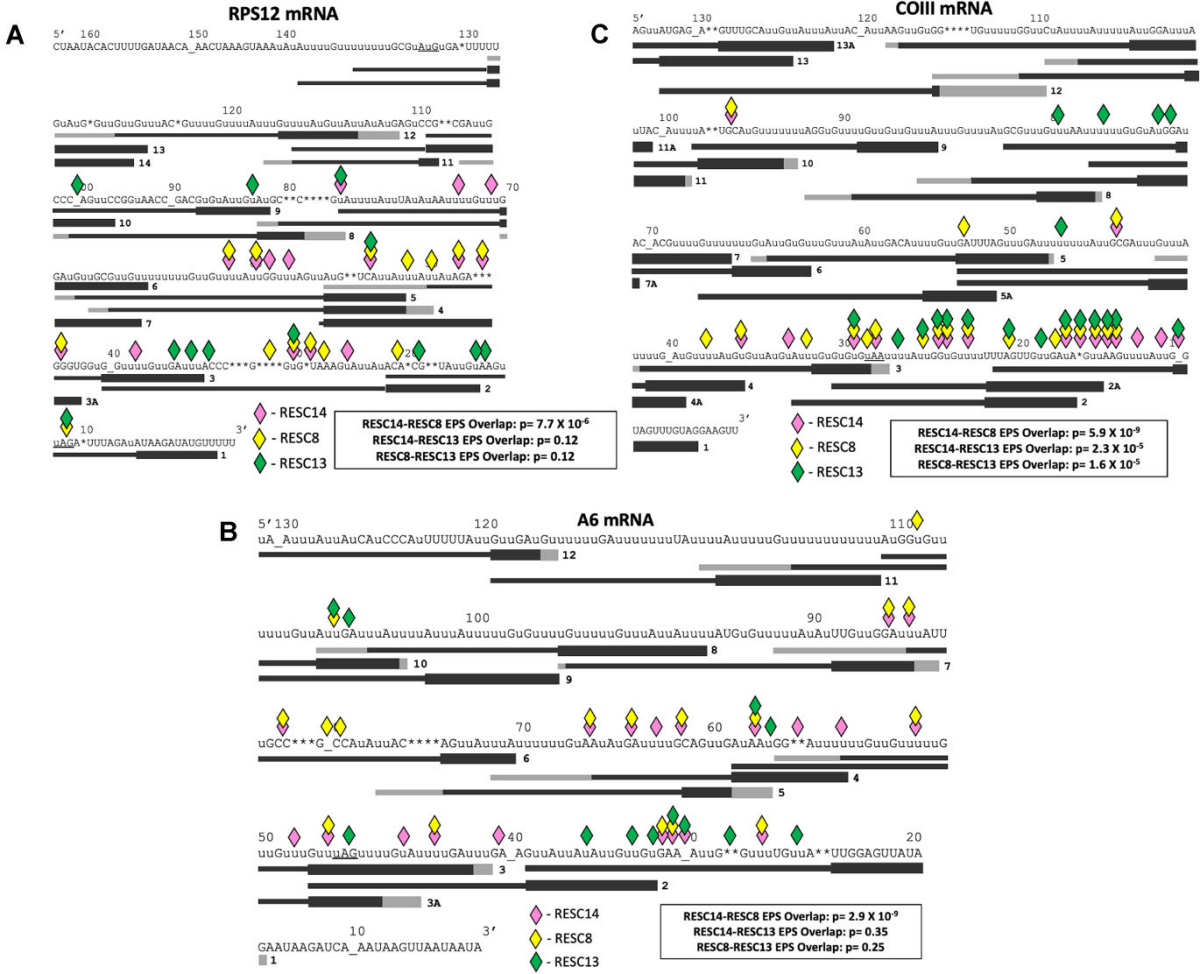
Comparison of editing defects in RESC14, RESC8, and RESC13 knockdown cell lines. RPS12 (**A**), A6 (**B**) and COIII (**C**) edited mRNA sequences with the exacerbated pause sites (EPSs; diamonds) that arise upon RESC14 (pink), RESC8 (yellow) and RESC13 (green) depletion. RPS12 data (A) was previously published for RESC13 (11), RESC14 (25) and RESC8 (24) RNAi cells. A6 (B) and COIII (C) EPSs for RESC14 and RESC13 RNAi were previously published (32). Black bars below the sequences represent previously reported gRNAs (6), which are numbered in the 3′ to 5′ direction along the mRNA. gRNA anchors are depicted as the black bold lines, and grey regions denote range of variation of gRNA lengths across members of the same gRNA class. Uppercase U’s are encoded in the mitochondrial genome; lowercase U's represent inserted uridines during the editing process. Asterisks (*) denote sites where encoded uridines were deleted during the editing process. Underscores are shown for clarity in stretches of unedited sequence to align editing site numbers with the correct editing site. The start and stop codons for RPS12 and only the stop codons for A6 and COIII are underlined. Significance of the overlaps between EPSs in different cells lines as determined using a Fisher's exact test (11) are shown in boxes below each transcript.

Two separate reports from our lab showed that editing defects across the RPS12 mRNA that arise upon RESC14 depletion are similar to those that occur when RESC8 is depleted, but largely distinct from those in RESC13-depleted cells, highlighting the functional differences between organizers and non-organizers (24,25). Here, we collate these data into a single figure (Figure 1A) to clearly compare the editing defects that occur in RPS12 mRNA editing when RESC14, RESC8 or RESC13 are depleted. While the overlapping phenotype between RESC14 and RESC8 is striking, it was only observed on one small, pan-edited mRNA (RPS12; 325 nucleotides (nt) fully edited) (Figure 1A) and one small editing domain (CYb) (24,25). Thus, we wanted to determine if the functional overlap between RESC14 and RESC8 is conserved across the longer pan-edited transcripts, A6 (820 nt fully edited) and COIII (969 nt fully edited) and whether RESC14 and RESC8 RNAi phenotypes consistently differ from those in RESC13 RNAi cells. To do so, we used high-throughput sequencing (HTS) and analysis of the output with the TREAT algorithm (11,33).

HTS/TREAT analysis allows us to identify pauses in editing of specific mRNAs after knockdown of specific editing factors to help reveal their role in editing and determine if two proteins function similarly. In this procedure, a library of pre-edited, partially edited, and fully edited sequences are obtained by Illumina MiSeq and then aligned by their editing sites (ES) using TREAT. An ES is any site between two non-U nucleotides. ESs are numbered 3′ to 5′, the general direction in which editing progresses. We can further analyze editing progression by defining the editing stop site, which is the 5′ most ES after contiguous canonical editing. To compare mRNA populations, we determine editing stop sites that are significantly overrepresented in a given knockdown cell line compared to uninduced controls (*P*_adj_ < 0.05), which are termed exacerbated pause sites (EPS) if they are significant in both biological replicates (11). Thus, an EPS is where correct editing pauses more often in cells depleted of a specific editing factor, allowing us to evaluate the step in editing progression in which that protein functions. Figure 1A illustrates the statistically significant overlap between RPS12 EPSs in RESC14 and RESC8 knockdown cells (*P* = 7.7 × 10^−6^) and suggests functional cooperation between these factors during editing progression. This significance is not observed when RESC14 and RESC13 RNAi EPSs are compared (*P* = 0.12), nor when RESC8 and RESC13 RNAi EPSs are compared (*P* = 0.12), indicative of somewhat distinct functions between RESC14/8 and RESC13. Comparison of these data with published EPS overlap data further inform the overlap of RESC factor functions. Previous studies reported the EPSs that arise on RPS12 mRNA after RESC2, RESC10, RESC11A and RESC12A RNAi (11,23), and Dubey *et al.* calculated the significance of pairwise EPS overlaps between these seven RESC factors (23). RESC10 EPSs significantly overlap with all RESC factors tested except RESC12A, consistent with an upstream function of RESC10, while RESC12A EPSs did not significantly overlap those of any other RESC proteins. Apart from RESC10, the only other factor with which RESC14 RNAi overlapped was RESC11A RNAi (*p* = 0.01), albeit this overlap is not as significant as the RESC14-RESC8 EPS overlap. RESC8 EPSs only overlapped those in the RESC14 and RESC10 knockdowns. Thus, the comparison of EPSs that arise on RPS12 mRNA after numerous RESC factor knockdowns further supports the specificity and functional cooperation between RESC14 and RESC8.

Using the strategy described above, we analyzed EPSs in A6 and COIII mRNAs that occur in response to RESC8 RNAi for comparison to previously reported EPSs in the same transcripts from RESC14 and RESC13 RNAi cells (32). Since the editing domains of A6 and COIII mRNAs are too large to be sequenced by MiSeq, only the 300 nt 3′ region of each transcript was sequenced. This means that ‘fully’ edited A6 refers to mRNA intermediates that are fully edited up to the forward primer (31). Due to sequencing discrepancies, ‘fully’ edited COIII refers to sequences where the canonical edited sequence matches up to ES 109, which is five editing sites 3′ of the true 5′ end of the amplicon (32). Libraries were generated for two induced RESC8 RNAi cell replicates, and these were compared to the two corresponding uninduced samples as well as to five PF 29–13 cells from another study (thus, compared to seven control samples total) (31). RESC8 EPSs were calculated and compared to the previously published EPSs that arise on A6 and COIII mRNAs upon RESC13 and RESC14 knockdown (Figure 1B and C) (32). Here, we find that RESC14 and RESC8 EPSs significantly overlap on A6 mRNA (*P* = 2.9 × 10^−9^), whereas RESC14 and RESC13 EPSs (*P* = 0.35) and RESC8 and RESC13 EPSs (*P* = 0.25) do not (Figure 1B). When analyzing the EPSs on COIII mRNA, we again calculate a significant overlap between the RESC14 and RESC8 EPSs (*P* = 5.9 × 10^−9^). We also observe a significant overlap between the RESC14 and RESC13 EPSs (*P* = 2.3 × 10^−5^), and RESC8 and RESC13 EPSs (*P* = 1.6 × 10^−5^) on COIII mRNA, which may in part reflect the overall large number of EPSs observed in the gRNA-1 and gRNA-2 directed regions of COIII mRNA (31) (Figure 1C). From these data, we conclude that there are conserved overlapping RESC14–RESC8 phenotypes on multiple pan-edited transcripts.

**Figure 2.**
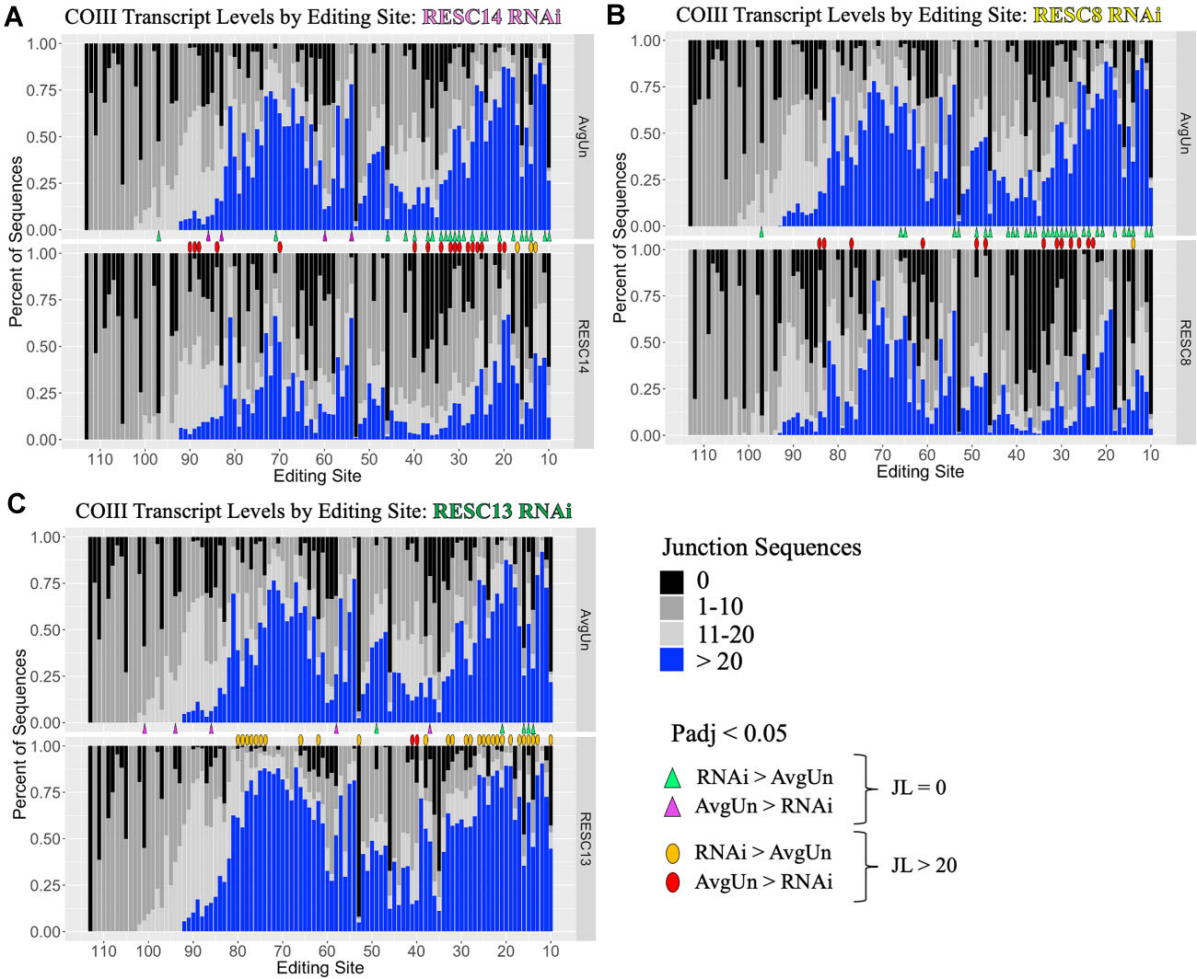
Effect of RESC14, RESC8, or RESC13 knockdown on junction lengths. The percent of sequences with junction lengths (JL) of 0 (black bars), 1–10 (light grey bars), 11–20 (dark grey bars), and greater than 20 (blue bars) at each editing site across COIII mRNA for RESC14 (**A**), RESC8 (**B**) and RESC13 (**C**) RNAi cells, compared to control samples. Triangles represent editing sites where there is a significant change in JL 0 sequences (black bars) between uninduced and induced cells (*P*_adj_ < 0.05); green triangles: RNAi is greater than uninduced cells, purple triangles: uninduced is greater than RNAi cells. Ovals represent editing sites where there is a significant change in JL greater than 20 editing sites (blue bars) between uninduced and induced cells (*P*_adj_ < 0.05); yellow ovals: RNAi is greater than uninduced cells, red ovals: uninduced is greater than RNAi cells.

Because COIII mRNA differed slightly from RPS12 and A6 mRNAs in that EPSs arising from RESC14/8 and RESC13 RNAi showed significant overlap, we wanted to compare editing defects in RESC14, RESC8 and RESC13 RNAi cells using junction length analysis. Sequences 5′ of an EPS can either be pre-edited or comprise a mis-edited sequence, called a junction. Junction analysis provides insight into specific editing defects that arise after editing factor knockdown. TREAT can define the lengths and sequences of junctions and scores a sequence with completely pre-edited sequence 5′ of an EPS having a junction length (JL) of 0. We analyzed the COIII mRNA population in RESC14, RESC8 and RESC13 RNAi samples, as well as in their uninduced counterparts and 29–13 controls, and characterized sequences at each editing stop site as having JL of 0, 1–10, 11–20 and > 20 (Figure 2). Many junctions are between 1–20 ESs long, as that is the approximately span of one gRNA. Junctions that are longer than 20 ESs likely arise through mRNA misfolding, which would direct editing more 5′ than expected based on gRNA sequence, or through extensive use of non-cognate and/or multiple gRNAs. Sequences that have a junction length of 0 represent a block in the 3′ to 5′ progression of canonical editing and a lack of junction formation.

When comparing RESC14 RNAi cells to control samples, we identified many sites where JL > 20 (blue bars) sequences significantly decrease (Figure 2A; red ovals) and JL = 0 sequences (black bars) significantly increase in the knockdown (Figure 2A; green triangles). This finding suggests that RESC14 is needed for the mis-editing progression that characterizes junctions. When comparing the phenotype of RESC14 RNAi to that of RESC8 RNAi, we observe a similar pattern: JL > 20 sequences decrease (Figure 2B; red ovals), while JL = 0 sequences increase with RNAi (Figure 2B; green triangles). Thus, RESC14 and RESC8 have a similar phenotype regarding junction formation. In contrast, in RESC13 RNAi cells, JL > 20 sequences increase (Figure 2C; yellow ovals), whereas JL = 0 sequences are relatively unchanged (Figure 2C; equal presence of green and purple triangles) when compared to uninduced samples. This junction length analysis shows that even though RESC13, RESC14 and RESC8 pause at similar sites on COIII mRNA (Figure 1C), the nature of the sequences 5′ of the pauses differ between RESC14/8 and RESC13. Similar junction length patterns were observed on A6 and RPS12 mRNAs (Supplemental Figures S1 and S2). Together, these data indicate that RESC14 and RESC8 have a related function during editing progression that is distinct from that of RESC13.

### RESC14 is needed for incorporation of a subset of RESC factors into large complexes

**Figure 3.**
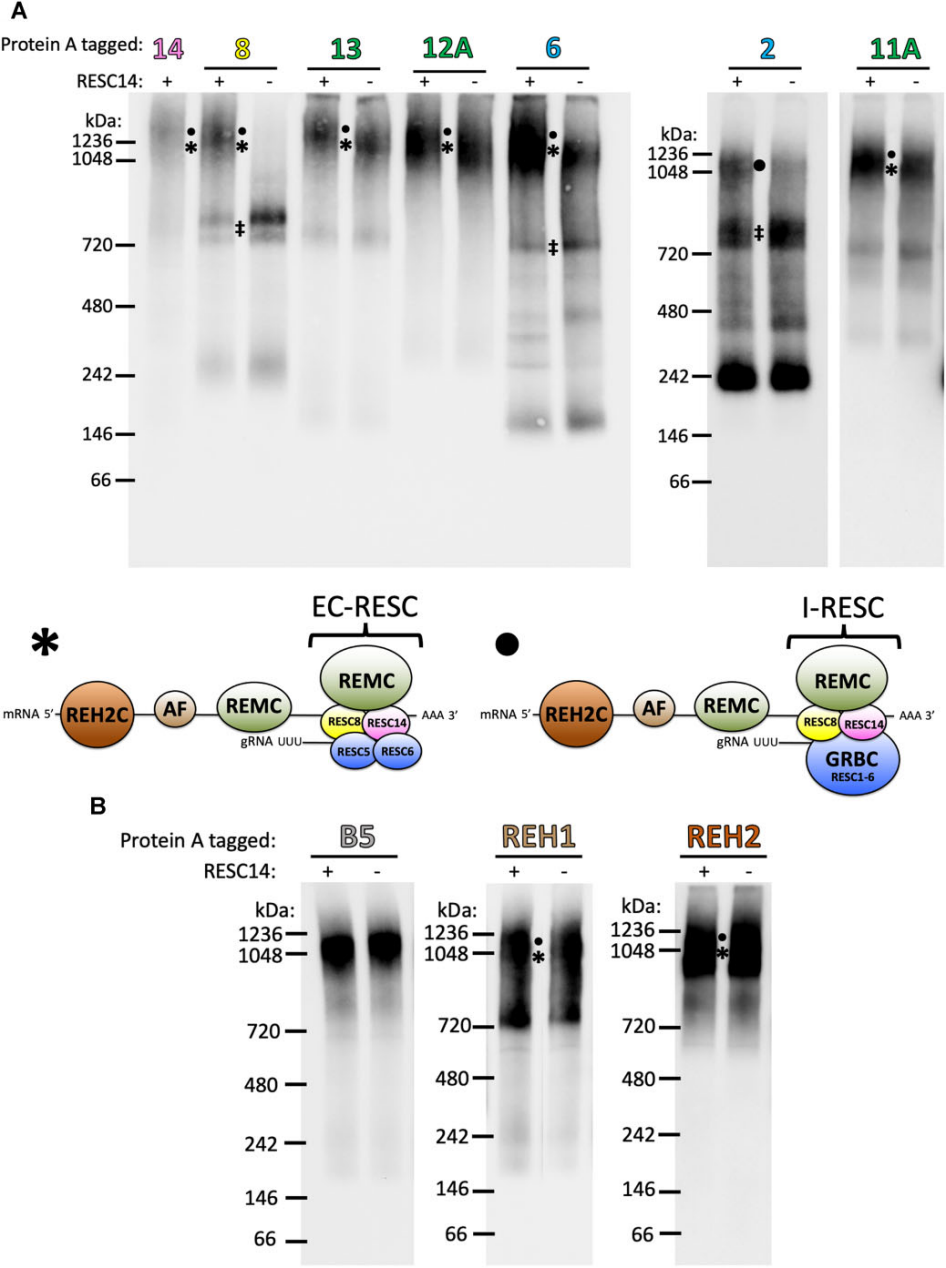
RESC14 RNAi results in impaired incorporation of specific RESC factors into large complexes. Blue native PAGE analysis of mitochondrial extract from cell lines containing Protein A-tagged proteins in the presence and absence of RESC14. (**A**) Blue native PAGE of MHT-tagged RESC14, RESC8, RESC13, RESC12A, RESC11A and PTP-tagged RESC6 and RESC2. (*) represents the complex containing the editing-competent form of RESC (EC-RESC). (**•**) represents the complex containing an intermediate form of RESC, prior to it becoming editing-competent (I-RESC). All RESC proteins tested, except for RESC2, predominately contain EC-RESC as part of the 1050–1250 kDa complex, but this complex also contains a small population of I-RESC (* and small **•**). Note that the vertical positioning of the * and small **•** symbols does not indicate their distinct sizes, but rather depicts that they are both components of the heterogenous large complex. The 1050–1250 kDa complex for RESC2 only contains I-RESC (large **•**). The bottom of (A) depicts the predicted proteins/RNA found in the large complexes denoted with a (*) and/or a (**•**). AF, accessory factors. The protein/RNA depiction of the complexes denoted with a (‡) are found in Figure 6B. The space between the RESC2 and RESC11A gels indicate different exposures from the same gel. (**B**) Blue native PAGE of non-RESC holoenzyme components MHT-tagged KREPB5, KREH1 and KREH2. PAP was used in western blotting to detect the Protein A region of each tagged protein. Blots are representative of three biological replicates.

To probe the mechanism by which RESC14 acts as an organizer during editing progression and begin to understand how its function overlaps that of RESC8, we analyzed the impact of RESC14 on the incorporation of editing holoenzyme components and accessory factors into multiprotein complexes. To this end, we generated numerous cell lines with editing factors tagged at a chromosomal locus with Protein A (either MHT or PTP tags), allowing us to visualize all protein complexes separated on the same blue native PAGE gel with the PAP reagent. We also made the corresponding cell lines with doxycycline-inducible RESC14 RNAi to investigate RESC14 function. We validated the cell lines by western blot (Supplemental Figure S3) and confirmed that the tags caused little or no growth defect (Supplemental Figure S4). To first understand the functional overlap between RESC14 and RESC8, we analyzed whether RESC14 and RESC8 form similar sized complexes and determined if RESC14 impacts the incorporation of RESC8 into multiprotein complexes. On blue native PAGE, RESC14 formed a single complex ranging from ∼1050 to 1250 kDa (Figure 3A). For RESC8 in the presence of RESC14, we also observed a complex of a similar size, as well as three smaller complexes of approximately 800, 750 and 300 kDa (note that the 750–800 kDa complexes were sometimes not well resolved, and so are denoted with one symbol, ‡; see Figure 5A). When RESC14 was depleted by RNAi, the large RESC8 complex disappeared, while the three smaller complexes accumulated (Figure 3A). Thus, we conclude that RESC14 is needed for the incorporation of RESC8 into a large complex, highlighting a mechanism for their functional interdependence.

We next asked how RESC14 affects the ability of other RESC factors to assemble into large multiprotein complexes, including components of both the REMC and GRBC modules. We looked at the complex formation of three well characterized REMC proteins (RESC11A, RESC12A and RESC13). RESC13, RESC12A and RESC11A also formed a large complex centering between 1050 and 1250 kDa in the presence of RESC14. However, unlike what was observed with RESC8, after RESC14 depletion we observed no reproducible change in the formation of these large REMC component containing complexes (Figure 3A), demonstrating that RESC14 is not needed for these proteins to assemble into large complexes. We also analyzed complex formation of GRBC components, RESC6 and RESC2, which again formed large complexes between 1050 and 1250 kDa. When RESC14 was depleted, these large complexes decreased in abundance, while smaller complexes accumulated for both tagged proteins (Figure 3A). Of the smaller accumulating complexes, some appeared to migrate similarly to the ‡ complex observed with RESC8. We also note that the decrease in RESC6 complexes of about 250 and 300 kDa after RESC14 RNAi was reproducible, suggesting these small complexes rearrange into 200, 400 and/or 700 kDa complexes when RESC14 is depleted. We observed that the vast majority of RESC2 is not in a large complex but is rather in smaller complexes, consistent with a previous report (21). The reduction in the largest RESC6- and RESC2-containing complexes and concomitant increases in smaller complexes is similar to, although not as dramatic as, what was observed for RESC8 when RESC14 is depleted. Thus, RESC14 also facilitates incorporation RESC6 and RESC2 into large multiprotein complexes.

To better understand how RESC14 affects holoenzyme dynamics, we determined whether RESC14 is needed for incorporation of non-RESC protein into complexes. Cell lines harboring tagged holoenzyme components KREPB5 (a RECC component) or KREH2 (an REH2C component), or the accessory factor KREH1 in the RESC14 RNAi background were generated (Supplemental Figures S3 and S4). For all three proteins, there was no visible change in complex formation after RESC14 RNAi, indicating that RESC14 is dispensable for RECC, KREH2 and KREH1 incorporation into large complexes (Figure 3B). We consistently observed that the KREH1-containing complexes formed more of a smear than complexes formed with other proteins analyzed, suggesting that KREH1 complexes are relatively less stable and causes them to disassociate in the gel. In conclusion, these analyses demonstrate that RESC14 is needed for incorporation of RESC8, RESC6 and RESC2 into multiprotein complexes. Based on our currently understanding of RESC composition and structure (2,21), RESC6 and RESC2 are most likely joining the large complex as components of GRBC. By contrast, RESC14 is dispensable for the incorporation of REMC (RESC13, RESC12A and RESC11A) and those non-RESC components tested, into large complexes.

We next attempted to perform tandem affinity purification of RESC protein-containing complexes to determine their protein compositions; however, the complexes were unstable and disassociated prior to or during native PAGE analysis, rendering us unable to elucidate their precise protein compositions. This further informs the idea that RESC is dynamic and not all proteins are stably associated *in vivo*. Nevertheless, consideration of RESC structures determined through cryo-EM, as well as our general understanding of editing proteins’ functions, is highly informative regarding the probable composition of the large complexes observed by native gel analysis. When first considering the RESC factor constituents of the large complexes, we observe that for each RESC factor tested, except for RESC2, the large 1050 to 1250 kDa complex is the most abundant complex (Figure 3A, complex *). Thus, we speculate that this complex contains of RESC5-14, as these proteins serve as the editing-competent form of RESC, here termed EC-RESC (a.k.a. RESC-B (21)), which might be expected to be the most abundant form of RESC. A small fraction of the total RESC2 population also migrates at a similar size, and RESC2 is not a component of EC-RESC. Thus, we envision the 1050 to 1250 kDa RESC2-containing complex as an intermediate complex, termed here I-RESC (Figure 3A, complex **•**), that forms following the joining of GRBC, REMC, and organizer proteins but before RESC1-4 dissociate. I-RESC then likely makes a minor contribution to the signal between 1050 to 1250 kDa observed with the other RESC proteins as well (Figure 3A, large complex denoted with both * and **•**). The limitations of resolution of blue native PAGE, as well as the contributions of both size and shape to complex mobility in these gels, likely contribute to the inability to clearly distinguish EC-RESC from I-RESC containing complexes by size. Thus, with regard to RESC composition, we hypothesize that all the large 1050 to 1250 kDa complexes formed with each RESC protein except for RESC2, contain primarily EC-RESC, with a small fraction being I-RESC (Figure 3A, large complex denoted with both * and **•**). For the RESC2 containing complex, we envision it as only comprising I-RESC (Figure 3A, complex **•**).

As aforementioned, when RESC14 is depleted, the abundance and mobility of the large REMC protein complexes do not change (Figure 3A). However, if we hypothesize that this large complex comprises similar complexes for each RESC factor tested, we would expect to see the 1050 to 1250 kDa complexes formed with REMC proteins decrease in abundance upon RESC14 RNAi as observed with RESC14, RESC8, RESC6 and RESC2. The REMC proteins are components of EC-RESC and I-RESC, but since a shift in the large complex formation is not observed, this suggests that the 1050–1250 kDa complexes contain multiple additional factors (Figure 3A, bottom), such that loss of the one GRBC module upon RESC14 RNAi only results in a small shift in size that is not resolved well by blue native PAGE. Candidates for additional factors include the association of multiple REMC proteins on pre-edited portions of mRNAs, which would be consistent with the association of REMC proteins primarily with pre-edited mRNA by *in vivo* UV cross-linking studies (21). We also observe no visible change in complex formation for RECC, KREH2 and accessory factor, KREH1 containing complexes when RESC14 is knocked down (Figure 3B). As RECC runs at ∼1100 kDa, in blue native PAGE (41), the large complex visualized for KREPB5 is most likely RECC itself. By contrast, given their known associations with RESC (2), it is likely that KREH2 and other editing accessory factors, including KREH1, are components of the same large complexes, or assembly of heterogeneous complexes, in which the RESC proteins are found (Figure 3A, bottom). Overall, the data in Figure 3 support a model in which complexes of approximately 1050 to 1250 kDa contain a large portion of EC-RESC and a small proportion of the I-RESC intermediate. Additionally, a mix of multiple REMCs, KREH2, and editing accessory factors including KREH1 likely contribute to the large size of these complexes. In summary, Figure 3 demonstrates the critical role of RESC14 in assembly of heterogeneous RESC-containing 1050–1250 kDa complexes.

### RESC8 is not essential for incorporation of RESC6 and RESC14 into large complexes

**Figure 4.**
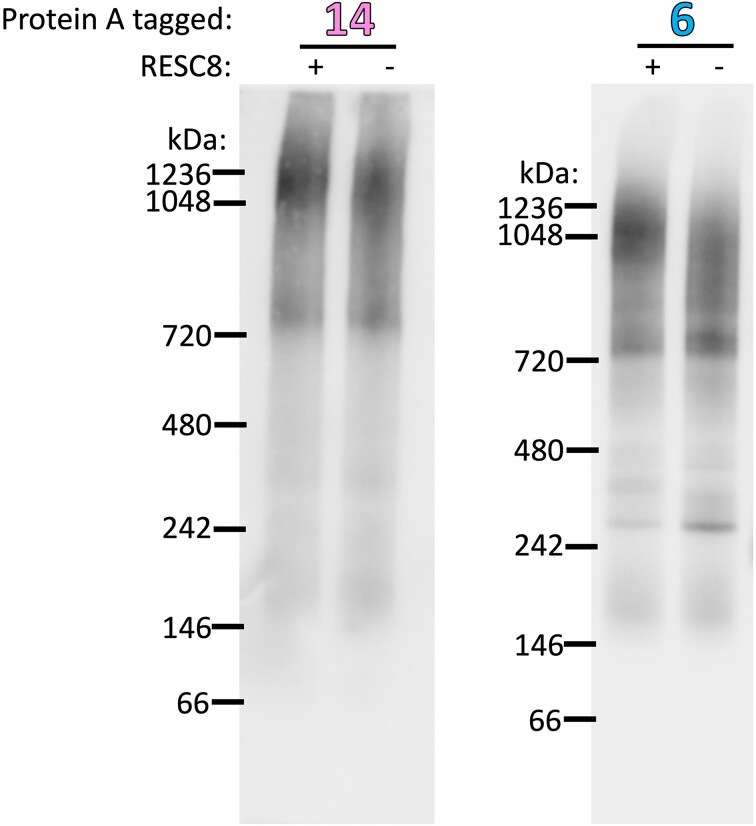
RESC8 RNAi has a minimal effect on RESC14 and RESC6 incorporation into large complexes. Blue native PAGE analysis of MHT-tagged RESC14 and PTP-tagged RESC6 from mitochondrial extracts in the presence and absence of RESC8. PAP was used in western blotting to detect the protein A region of the tag. Blots are representative of three biological replicates.

As shown in Figure 3A, when RESC14 is depleted, RESC8 and RESC6 are not efficiently incorporated into large complexes. We next wanted to test whether RESC14 and RESC8 are mutually necessary for large complex formation and whether changes in RESC6-containing complexes are due to lack of RESC14 and/or lack of RESC8. To answer these questions, we generated cell lines containing tagged RESC14 and RESC6 in the RESC8 RNAi background (24) (Supplemental Figures S3 and S4) and performed blue native PAGE analysis followed by detection of complexes with the PAP reagent. We observe that when RESC8 is depleted, there is no change in RESC14 complex formation (Figure 4). In the case of RESC6, we observed a reduction in the large 1050–1250 kDa complex when RESC8 is depleted and a smear below the large complex, suggesting destabilization, but not complete dissociation, of the large complex upon RESC8 RNAi. (Figure 4). This pattern is distinct from that observed upon RESC14 depletion, where there clear decrease in large complex formation indicated dissociation of RESC6-containing large complexes (Figure 3A). These experiments show that RESC8 is dispensable for RESC14 complex formation. Moreover, the absence of RESC8 only modestly affects RESC6 incorporation into the large complex. Thus, the dramatic effects of RESC14 on complex formation (Figure 3A) cannot be entirely attributed to downstream effects on RESC8.

### RESC14 is required for efficient association of RESC8 and GRBC with a large RNA-containing complex

**Figure 5.**
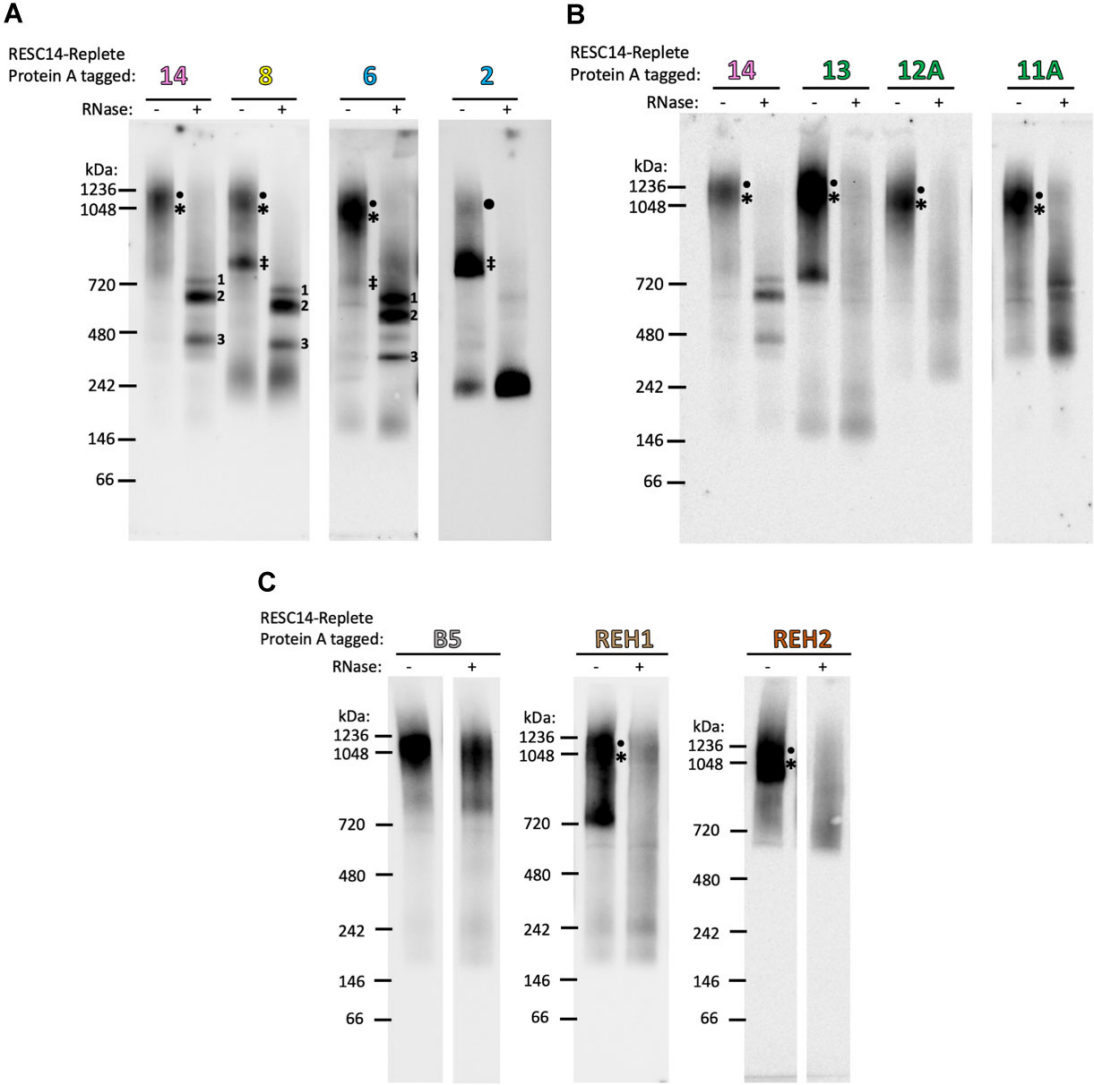
Large RESC complexes contain RNA. Blue native PAGE analysis of mitochondrial extract from cell lines containing Protein A-tagged editing factors in the presence and absence of RNase treatment for RESC14-replete cells. (**A**) Blue native PAGE of MHT-tagged RESC14 and RESC8, and PTP-tagged RESC6 and RESC2. (**B**) Blue native PAGE of MHT-tagged RESC11A, RESC12A and RESC13. **(C)** Blue native PAGE of MHT-tagged KREPB5, KREH1 and KREH2. Complexes **1–3** in (A) likely represent the same RESC assembly or disassembly intermediates between RESC14, RESC8 and RESC6. The (*, EC-RESC) and (**•**, I-RESC) complex components are described in Figure 3A. Note that the vertical positioning of the * and small **•** symbols does not indicate their distinct sizes, but rather depicts that they are both components of the heterogenous large complex. The (‡) complex components are described in Figure 6B. PAP was used in western blotting to detect the Protein A region of each tagged protein. The spaces between gels in (A) and (B) indicate different exposures from the same gel, where the spaces between gels in (**C**) represent excised lanes from the same gels. The left panel of KREPB5 in (C) is the same blot depicted in Figure 3B. Blots in (A) and (B) are representative of three biological replicates; blots in (C) are representative of two biological replicates.

To further assess the content of the large complexes described above, we determined which complexes contain RNA by comparing their gel migration patterns in the presence and absence of RNase treatment. We harvested lysates of cells harboring the tagged RESC factors described above either in the presence of RNA (lysate treated with RNase inhibitor) or absence of RNA (lysate treated with an RNase cocktail). These lysates were subjected to blue native PAGE analysis followed by detection with the PAP reagent. When analyzing the complexes containing RESC14, RESC8 and GRBC (RESC6 and RESC2), we observed that all the large complexes disappear, while smaller complexes accumulate after RNase treatment (Figure 5A). Thus, large complexes formed with RESC14, RESC8, RESC6 and RESC2 contain RNA (Figure 5A, complexes * and **•**). Under RNase-treated conditions, RESC14, RESC8 and RESC6 form three similar sized, smaller complexes around 720, 600 and 450 kDa (Figure 5A, complexes 1–3), suggesting that these three proteins can be found together in non-RNA containing complexes. We next analyzed complexes containing REMC components. Figure 5B shows the effects of RNase treatment on complexes harboring tagged RESC13, RESC12A and RESC11A. In each case, RNase treatment leads to disappearance of the large 1050–1250 kDa complexes, while no clear smaller complexes accumulate, with the exception of RESC11A for which some complexes of similar sizes to complexes 1–3 and smaller were observed within the smear. As a control, we analyzed tagged RESC14 untreated or treated with RNase on the same gel and, as expected, distinct smaller complexes 1–3 accumulated in RNase-treated samples (Figure 5B). The pattern observed with RNase-treated REMC proteins is unlike what was observed for RESC14, RESC8 and GRBC (Figure 5A) and indicates that, in general, the REMC proteins are unable to form stable complexes in the absence of RNA. We next analyzed how RNase treatment impacts the formation of complexes containing KREPB5, KREH1 and KREH2 (Figure 5C). Similar to what we observed with the REMC proteins, RNase treatment of KREH1 and KREH2 resulted in the disappearance of the large 1050–1250 kDa complexes with little to no accumulation of smaller complexes. By contrast, the large complex formed with RECC component, KREPB5, is still present after RNase treatment (Figure 5C), consistent with this large complex comprising solely RECC. The similar RNase sensitivity of KREH1- and KREH2-containing complexes (Figure 5C) to those containing RESC factors (Figure 5B) further supports our model in which the large 1050–1250 kDa complexes are heterogeneous and likely contain EC-RESC and mixtures of multiple REMC modules, KREH2 and accessory factors (with the exception of RESC2-containing complexes, which comprise I-RESC) (Figure 3A, bottom). Together, the data presented in Figures 3 and 5 establish that RESC14 is needed for efficient association of RESC8 and GRBC with a large, RNA-containing complex.

Figure 5 shows that the large complex contains RNA; thus, we speculate the large complex contains gRNA and mRNA (Figure 3A, bottom). Since we do not know the editing extent of the mRNA in this complex, its relative size would be ∼1050–1250 kDa, with the low end representing a pre-edited mRNA component and the high end representing a fully edited mRNA component. The three similarly sized, smaller complexes around 720, 600 and 450 kDa that contain RESC14, RESC8 and RESC6 after RNase treatment likely represent intermediates of RESC protein remodeling, possibly during removal of a fully utilized gRNA (Figure 5A, complexes 1–3). Since RESC14, RESC8 and RESC6 are components of complexes 1–3, these complexes may comprise or be related to the previously described RESC-C (which contains RESC5-8, 10 and 14) determined through cryo-EM (21).

**Figure 6.**
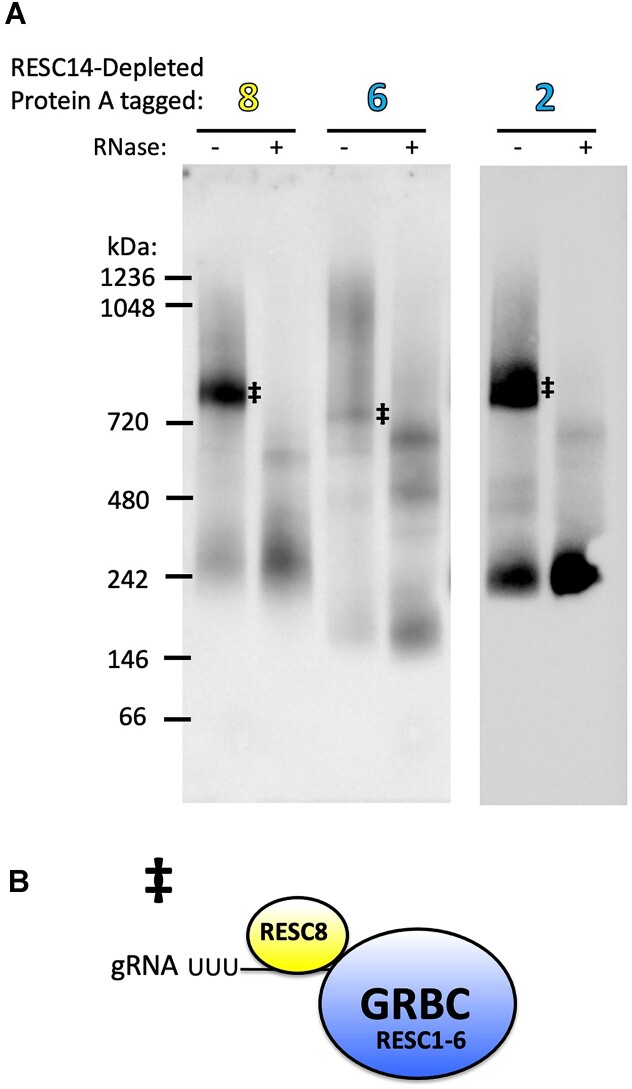
Identification of RESC assembly intermediates. (**A**) Blue native PAGE analysis of cell lines expressing MHT-tagged RESC8, RESC6 and RESC2 with RESC14 depleted in the presence and absence of RNase treatment. (**B**) Depicted are the predicted protein/RNA components of the (‡) complexes. PAP was used in western blotting to detect the Protein A region of each tagged protein. The space between gels represents different exposures from the same gel. Blots are representative of three biological replicates.

Having shown that the large 1050–1250 kDa complex contains RNA, we next asked whether any smaller RESC complexes contain RNA. As shown in Figures 3A and 5A with the **‡** symbol, in cells replete for both RESC14 and RNA, tagged RESC8, RESC6, and RESC2 all consistently formed a complex of between 720–750 kDa (sometimes appearing as a doublet, referred to here as a single complex for simplicity). Given that proteins run in native PAGE as a function of both size and shape, and tagging different proteins in a complex might differentially alter complex mobility, these 720–750 kDa bands could represent the same or similar complexes. When RESC14 is depleted by RNAi, the abundance of this 720–750 kDa complex increases (Figure 3A). To better define the 720–750 kDa complex, we first depleted cells harboring tagged RESC8, RESC6 or RESC2 of RESC14 by RNAi to increase the abundance of this complex (Figure 6). Next, we subjected lysates from these cells either to an RNase inhibitor or an RNase cocktail and monitored RESC protein migration by blue native PAGE with the PAP reagent. When treated with RNase, each of these complexes disappears, while even smaller complexes accumulate (Figure 6A, complex ‡). These findings, thus, suggest that RESC8, RESC6, and RESC2 form a small complex with one another that does not contain RESC14 but does include RNA. Based on the relatively small size of the complex (GRBC plus RESC8 is ∼430 kDa) and the presence of gRNA-stabilizing protein RESC2 (42,43), we hypothesize that the RNA component of these small complexes is gRNA. We note that a complex containing GRBC, RESC8 and a gRNA would only be expected at ∼460 kDa, but the complex observed by blue native PAGE is ∼720–750 kDa. This could mean that the size, shape or tag component of the complex leading to slower migration than expected in the native gel. Another likely explanation is that additional RESC proteins that were not tagged and tested here, such as RESC7, RESC9 and RESC10 are components of this complex. We also note a previous report that REH2C may be associated with a similar complex (21), bringing the expected size to ∼790 kDa. We do observe a KREH2-containing complex of ∼750 kDa by blue native PAGE (Figures 3B and 5C); however, this complex does not increase in abundance after RESC14 RNAi, so this is not likely the 720–750 kDa complex under analysis here. Overall, to this point, our data support a model in which RESC14 recruits a smaller ribonucleoprotein complex (RNP) containing RESC8, GRBC and a gRNA (Figure 6B, complex ‡) to a larger complex that contains RNA. We envision that this recruitment ultimately leads to the formation of EC-RESC associated with mRNA, gRNA, and additional mRNA-associated factors as described above (Figures 3 and 5, complex *).

### RESC14 is needed for stable RESC assembly and RESC–RECC interactions

Experiments described to this point focus on distinct editing factors and their respective complex formation in cell lysates. To both get a broader view of protein–protein interactions modulated by RESC14 and to evaluate these interactions *in vivo*, we turned to TurboID proximity labeling followed by mass spectrometry. We TurboID-tagged RESC6 and RESC8 at their endogenous loci in the RESC14 RNAi background (Supplemental Figure S5) to gain insight into how RESC14 impacts specific protein interactions with a GRBC component (RESC6) and a RESC organizer (RESC8). Parental strain 29–13 cells were used as a negative control. We isolated biotinylated proteins from mitochondrial extracts using streptavidin beads followed by mass spectrometry analysis. We identified 791 proteins in at least one replicate from all conditions, with 495 proteins enriched at least 2-fold with RESC6 or RESC8, when normalized to parental 29–13 cells (Supplemental Tables S6-8).

First, we asked which mitochondrial RNA editing and processing proteins were in proximity to RESC6 and RESC8 and how their proximity changed after RESC14 depletion. We identified 26 proteins within the RESC6-TurboID and RESC8-TurboID samples (Table 1). Of these 26 proteins, six proteins exhibited significant proximity changes with RESC6 and two proteins with RESC8 after RESC14 depletion (Table 1, pVal columns; yellow). RECC constituents KREN1, KREPA1, KREPA2, and KREPB8 decreased in proximity to RESC6 (Table 1, Log_2_ ratio columns; green), while KREN1 and KREPA2 significantly decreased in proximity to RESC8 as well. A Log_2_ ratio value could not be determined for other RECC components, KREX1 and KREN3, in the RESC6-TurboID samples, since no peptides were identified in any replicate of the plus doxycycline (RESC14-depleted) samples. However, we observe peptides in all three replicates of the minus doxycycline (control) samples, indicating that the proximity of KREX1 and KREN3 to RESC6 also decreased when RESC14 was depleted. These data indicate that depletion of RESC14 and the subsequent RESC dissociation negatively impacts RECC-RESC interactions. By contrast, biotin labeling of RESC11A and RESC12 (REMC proteins) by RESC6-TurboID increased when RESC14 was depleted. Based on these data and native gel analysis in Figure 3A, we hypothesize that, without RESC14, GRBC undergoes continuous interactions with mRNA-bound REMC proteins but fails to maintain a stable association. Repeated transient GRBC-REMC interactions then lead to increased biotinylation of RESC11A and RESC12 by RESC6-TurboID.

**Table 1.** RNA editing proteins identified from TurboID with RESC6 and RESC8 and how RESC14 impacts their interactions

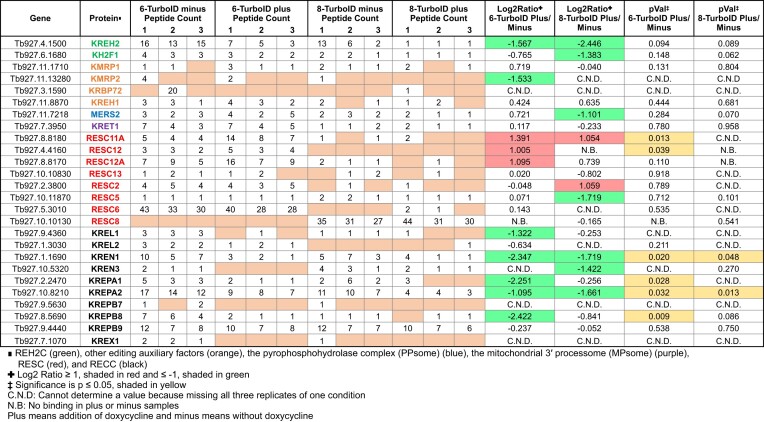

To identify other protein interactions controlled by RESC14 outside of our expected RNA editing and processing factors, we analyzed the proteins that had at least 2-fold enrichment with either RESC6 or RESC8 compared to parental 29–13 cells and exhibited a significant proximity change after RESC14 depletion. We identified 13 proteins with such properties (Table 2). Uncharacterized mitochondrial proteins Tb927.3.4210, Tb927.10.9280 and Tb927.4.3070 were highly enriched with RESC6 and RESC8, but only their interaction with RESC8 significantly changed after RESC14 depletion (Table 2). Interestingly, these three proteins were more enriched with RESC6 and RESC8 than was any RNA editing or processing protein (Table 1); thus, future studies investigating their functions could help reveal a potential role in the editing process. Together, TurboID biotinylation results demonstrate that RESC14 impacts numerous mitochondrial protein–protein interactions either directly or through its impact on proper RESC assembly. These data further support a model in which RESC14 impacts EC-RESC assembly and, in turn, EC-RESC assembly is needed for RESC–RECC association.

**Table 2.** Non-editing proteins that significantly change in proximity to RESC6 and RESC8 after RESC14 depletion

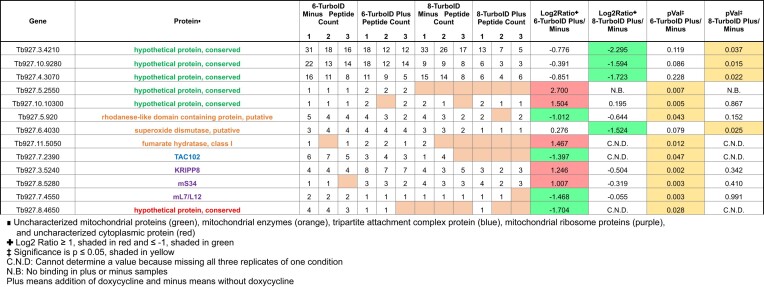

### RNA modulates RESC14 and RESC8 interactions with each other and other RESC proteins

**Figure 7.**
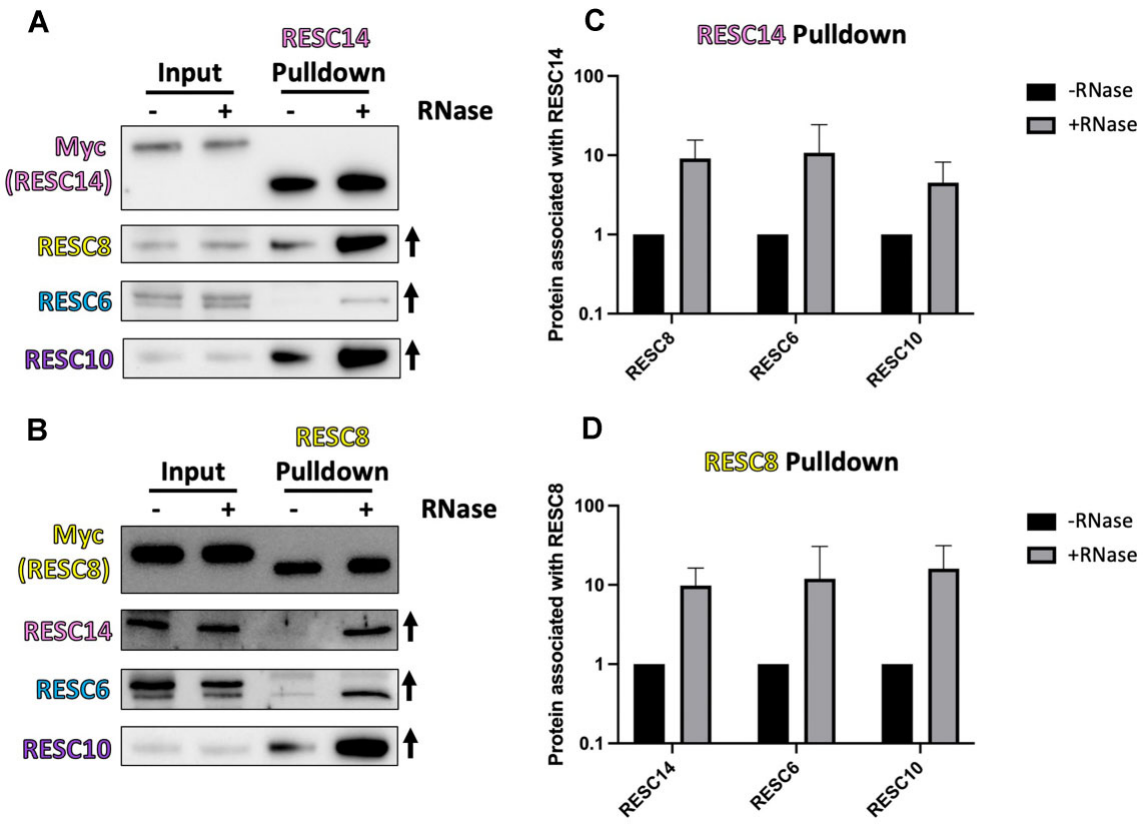
RESC14 and RESC8 interact more strongly with each other and with other RESC factors in the absence of RNA. Endogenously tagged RESC14-MHT (**A**) or RESC8-MHT (**B**) were affinity purified from cell extracts that were either RNase inhibited or RNase treated. Tagged proteins were pulled down using IgG beads and eluted off the beads using TEV protease cleavage. Input samples and TEV elutions were subjected to western analysis using antibodies to myc (to detect the pulled down protein) and other editing proteins. Arrows indicate the change in protein association with RNase treatment. (**C**) Is a quantification of (A), and (D) is a quantification of (B). These are representative blots of three biological replicates.

RESC14 and RESC8 are components of multiple RNPs (Figures 3–6) (21,24,25). We previously reported that association of RESC14 and a subset of RESC proteins, including RESC8, is inhibited by RNA to varying degrees; however, these studies were performed with overexpressed RESC14 (25). To better define the protein–protein and protein–RNA interactions within RESC RNPs consisting of RESC14, RESC8, RESC6 and RESC10, we isolated RESC14 that was endogenously MHT-tagged from lysates either treated with RNase cocktail or RNase inhibitor and performed western blots using antibodies against RESC8, RESC6 and RESC10. We reproduced the previously reported RNA inhibition of RESC14 interactions with RESC6 and RESC8, and we further showed that the RESC14-RESC10 interaction is also RNA-inhibited (Figure 7A and C). Since we are interested in understanding how RESC14 and RESC8 functions depend on each other during RESC dynamics, we next wanted to identify how RNA impacts RESC8’s interaction with these same RESC components. To do so, we performed an affinity purification with our endogenously MHT-tagged RESC8 cells in the presence and absence of RNase treatment. As expected, the RESC8-RESC14 interaction detected in this manner is RNA-inhibited (Figure 7B and D). Moreover, RESC6 and RESC10 also had RNA-inhibited interactions with RESC8, as these interactions increase with RNase treatment (Figure 7B and D). The similarity between RESC14 and RESC8 regarding RNA-dependent and RNA-inhibited interactions further supports their cooperative effect on RESC dynamics.

## Discussion

**Figure 8.**
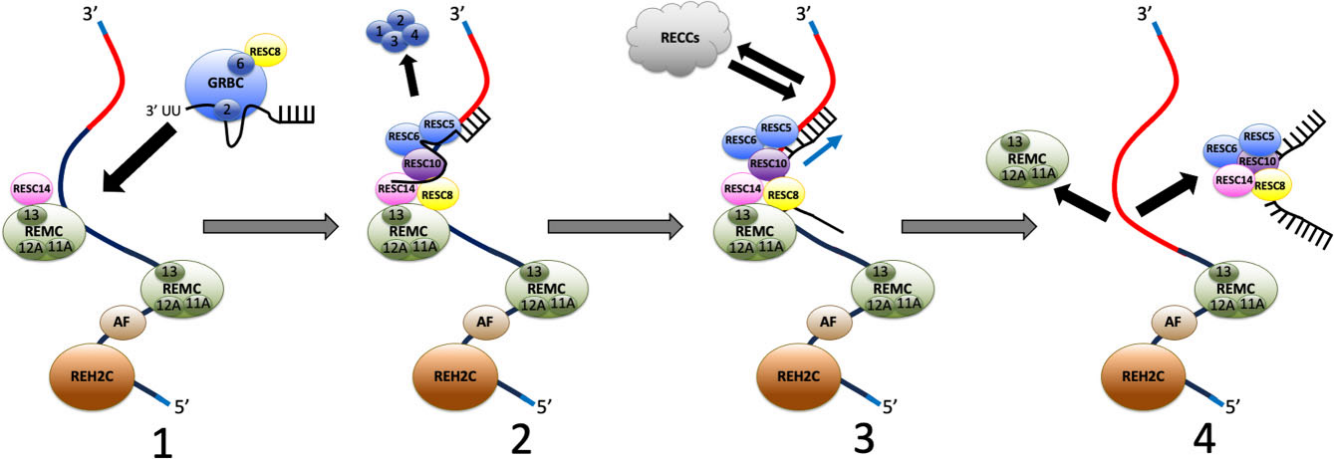
Working model for RESC14 and RESC8 function. See text for detailed description. Black arrows denote protein movement. Blue arrow denotes RNA movement. The red portion of the mRNA is fully edited, where the navy portion is pre-edited. Light blue ends of the mRNA designate the never-edited regions. AF, editing accessory factors.

RESC is a dynamic complex, composed of multiple modules and organizer proteins, that serves as the scaffold for U-indel RNA editing (15,22–25). It is responsible for coordinating interactions between the gRNAs, mRNAs and RECCs (2). A recent study using cryo-EM identified three RESC protein/RNA complexes whose compositions confirm that RESC’s function in editing requires multiple rearrangements (21), but how this remodeling from complex to complex is facilitated during editing progression and gRNA exchange is not known. In this study, we further characterize the functions of RESC organizer proteins, RESC14 and RESC8, in facilitating RESC dynamics. Previous studies showed that depleting either of these proteins leads to the disassociation of the REMC and GRBC modules of RESC, but no loss in integrity of either subcomplex (24,25). gRNA and mRNA interactions with RESC proteins were also disrupted with RESC14 and RESC8 RNAi, albeit in different ways (24,25). Here, we combine HTS and bioinformatic analysis with blue native PAGE analysis of RESC subcomplexes and TurboID based proteomics to develop a model of RESC dynamics, and the roles of RESC14 and RESC8 in this process (Figure 8).

In Step 1 (Figure 8), we envision that RESC8 associates with GRBC and gRNA, while RESC14 associates with REMC proteins on the mRNA. Using native PAGE, we identified small RNA-containing complexes of approximately 720–750 kDa, for RESC8, RESC6, and RESC2, that do not contain RESC14, as they accumulate upon RESC14 knockdown (Figures 3A, 5A and 6A, complex ‡). As these complexes migrate at a similar size between RESC8, RESC6 and RESC2, we envision that they are same, or similar, to each other. Due to the presence of the gRNA stabilizing protein, RESC2 (42,43), and the small size of the complex, a gRNA component seems likely. In support of this, cryo-EM also identified a structure containing GRBC proteins and gRNA (a.k.a. RESC-A) (21). The absence of RESC8 in this cryo-EM complex suggests that the RESC8-GRBC association is transient, and occurs just prior to assembly of GRBC into a larger complex. Since RESC14 is necessary for RESC8, RESC6 and RESC2 to assemble into large complexes (Figure 3A), we hypothesize that RESC14 is positioned on a larger complex, poised for association with RESC8. Supporting this model, RESC8 is not necessary for RESC14 to associate with a large complex.

Native PAGE shows that all tested RESC proteins are found in large RNA containing complexes between 1050 to 1250 kDa (Figures 3A and 5A). We speculate that this large complex forms when RESC8 and RESC14 are properly positioned with GRBC/gRNA and REMC/mRNA, respectively. Subsequently, GRBC and REMC associate, likely through the direct RESC8/RESC14 interaction that is observed by cryo-EM (21) (Figure 8, Step 1 to Step 2). Previous studies showed that GRBC and REMC are separate modules that interact in an RNA-dependent manner (15,22), and RESC14 is important for productive GRBC–REMC interactions (25), together supporting the model that these modules start out separate and are brought together via mRNA. Two lines of evidence reported here indicate that in the absence of either RESC14 or RESC8, GRBC and REMC attempt to interact, but the resulting complex is unstable. First, we observed by native PAGE that in RESC8 knockdowns, RESC6 struggles to form a large complex and instead forms a smear (Figure 4). Moreover, TurboID studies showed that in the absence of RESC14, interactions between RESC6-TurboID and REMC proteins, RESC12 and RESC11A, actually increase (Table 1). We interpret these data to mean that when RESC14 is depleted, the putative small GRBC/RESC8/gRNA complex consistently tries, but fails, to join REMC on the mRNA, causing more frequent RESC6-REMC interactions and resulting in increased biotinylation of RESC12 and RESC11A. HTS/bioinformatic analysis of editing intermediates supports an overlapping function for RESC8 and RESC14 during RNA editing (Figures 1 and 2), namely their possible shared roles in assembling GRBC and REMC into the editing competent complex, EC-RESC (Figure 8, Step 1 to Step 2). Both RESC8 and RESC14 knockdowns lead to numerous EPSs across pan-edited mRNAs and a dramatic increase in mRNAs lacking junctions, highlighting their functions in the 3′ to 5′ progression of both canonical and non-canonical editing. These data establish the critical importance of RESC8/RESC14’s facilitation of large RESC complex assembly, ultimately resulting in the formation of EC-RESC.

Initially, we envision that the entirety of GRBC (RESC1–6) will join REMC, forming I-RESC. This allows for the delivery of the gRNA to the mRNA. This intermediate complex may change quickly, since it was not identified by cryo-EM (21). Moreover, we find that a large complex containing tagged-RESC2 comprises a small percentage of total RESC2 (Figure 3A and 5A, complex **•**). After RESC14 helps bring the putative GRBC/RESC8/gRNA complex to REMC and the mRNA we hypothesize that, rather quickly, RESC1–4 leave the large complex and RESC10 enters, forming the editing-competent version of RESC, EC-RESC (Figure 8, Step 2). A rearrangement entailing RESC1-4 dissociation is consistent with a stable structure comprised of RESC5–14, gRNA and mRNA (RESC-B) that was identified by cryo-EM (21). As RESC10 is important for the interaction of RESC5/6 (23), it is likely that this protein joins to stabilize this protein interaction. Since tagged RESC10 is non-functional (23), we could not perform experiments to analyze RESC10-containing complexes. RESC10 may be a component of the predicted GRBC/RESC8/gRNA small complex, or join EC-RESC during or just after RESC1-4 dissociation. It is unlikely that RESC10 would be positioned initially with the REMCs, because RESC10 is the only RESC factor that exhibits minimal interaction with RESC13 (23). As each RESC factor tested by native PAGE, other than RESC2, forms the large 1050 to 1250 kDa complex abundantly, we hypothesize that the large complex contains EC-RESC, along with an mRNA and gRNA, plus additional proteins described below.

Within the heterogenous 1050–1250 complex, in addition to EC-RESC, we also speculate that there may be multiple REMC modules assembled on an mRNA (Figure 8). In this study, we tagged and followed REMC proteins RESC11A, RESC12A and RESC13, and thus we only indicate these specific proteins in our model (Figure 8). We note, however, that RESC9 has also been described as a REMC component (15), and that cryo-EM structures could be consistent with RESC7 being associated with REMC as well (21); the roles of RESC9 and RESC7 in RESC dynamics await future experiments. Native PAGE shows that when RESC14 is depleted, there is no apparent change in the mobility or abundance of large complexes formed with the REMC proteins tested (Figure 3A). Given that the 1050–1250 kDa RESC8, RESC6 and RESC2-containing complexes are reduced or absent in RESC14-depleted cells, we might also expect to see REMC protein-containing large complexes reorganize into smaller complexes on the native gel upon RESC14 depletion, as we hypothesize that the large RESC complexes visualized are similar to each other (Figure 3A). One explanation for the lack of such a shift is that there are multiple REMC modules in this complex, so that when one GRBC module is absent after RESC14 depletion, there is little to no apparent change in the size of the large complex, given the combined size of multiple REMCs/mRNA and the resolution of the native gel. We speculate that these multiple REMC modules are positioned upstream of active editing, on the 5′ pre-edited region of the mRNA (Figure 8). HTS/TREAT analysis of mRNAs in RESC13 RNAi cells revealed an increase in abnormally large junctions on multiple transcripts (Figures 2C, S1C and S2C) (11,32). Additionally, we previously reported that knockdown of either of the REMC proteins, RESC13 or RESC12/12A, leads to disjoined editing on RPS12 mRNA in which patches of edited sequence are observed far 5′ of the region being actively editing (11). Thus, when REMC proteins are depleted, some editing action is permitted further 5′ than expected. This finding indicates that REMC is important for constraining the region of active editing, which it may do through its positioning at multiple points on the pre-edited mRNA, 5′ of the active editing region (Figure 8) (11,32). *In vivo* UV cross-linking analysis demonstrated that RESC13 and RESC12/12A display a strong preference for pre-edited mRNA over fully edited mRNA (21), again supporting a model in which multiple REMC modules are positioned on the 5′ pre-edited mRNA region. In addition to multiple REMCs being constituents of the heterogeneous, large complex, we speculate that KREH2 and editing accessory factors (2), including KREH1, are also components of the 1050–1250 kDa complex. Native PAGE analysis shows that both KREH2 and KREH1 form a large complex that contains RNA, and this complex is unaffected after RESC14 depletion (Figures 3B and 5C). Because KREH2 and KREH1 exhibit a phenotype similar to that of the REMC proteins, we posit that these factors are positioned with the multiple REMC modules on the RNA, thus contributing to the heterogeneous large complex (Figure 8).

Once EC-RESC is formed and properly positioned on the mRNA, this presumably permits the RECCs to associate and catalyze U-indel editing (Figure 8, step 3). The gRNA–mRNA duplex is fed through RESC, protruding from RESC5/6, which allows the RECCs to bind the RNA and catalyze U insertion and deletion (Figure 8, step 3, blue arrow) (21). TurboID studies reported here show that RESC14 is important for allowing the RECCs to come in close proximity to RESC6 and RESC8 (Table 1). Thus, when RESC14 is not present, the RESC/mRNA is not in a permissive conformation for productive RECC association. This non-permissive conformation could be due to the initial improper joining of REMC and GRBC, or the disassembly of an unstable RESC complex lacking RESC14. Impairment of the RECC-RESC association in RESC14-depleted cells is consistent with bioinformatic analysis showing that RESC14 is needed for editing progression in general, whether for canonical editing or junction formation (Figures 1, 2A, S1A and S2A).

After the RECCs have generated a fully edited sequence throughout the length of a gRNA-directed block, it is likely that RESC disassembles and leaves the mRNA. This disassembly would be needed for recruitment of a new GRBC (RESC1-6)/gRNA complex. We envision that RESC5, RESC6, RESC14, RESC10, RESC8 dissociate from the mRNA along with the previously utilized gRNA (Figure 8, step 4). Consistent with such a disassembly intermediate, cryo-EM studies identified a complex containing RESC5-8, RESC10 and RESC14 (a.k.a. RESC-C) (21) and gRNA. We showed that RESC14 and RESC8 have RNA-inhibited interactions with each other, as well as RESC6 and RESC10 (Figure 7) (25). Thus, the protein components of this dissociated complex may interact more strongly with one another either after rearrangement or degradation of the gRNA. It is likely that the gRNA component of this complex is destined for destruction since a previous report showed that gRNAs are degraded after their use (15). Editing then continues with the subsequent gRNA, positioned within a GRBC module, recruited to the next REMC module already positioned on the mRNA through the combined actions of RESC14 and RESC8 (Figure 8, step 4).

The dynamic nature of RESC assembly is reminiscent of other facets of RNA biology such as pre-mRNA splicing. During spliceosome assembly, dynamic rearrangements allow for quality control and modulation of alternative splicing (44). Analogous functions can be envisioned during U-indel editing, where correct mRNA-gRNA pairing and positioning of RNA for productive RECC association must occur hundreds of times for complete editing of a single pan-edited mRNA. In addition, alternative editing of some transcripts has also been proposed and is likely to be regulated (45,46). Thus, checkpoints during RESC assembly may monitor and impact these aspects of the editing process. KREH2 interaction could be important during these steps, as this helicase has been implicated in both proofreading (19) and control of alternative editing (46). That RESC8 plays a critical role in RESC dynamics, as shown here, is likely related to its being comprised entirely of helical repeats that resemble ARM or HEAT repeats. Both ARM and HEAT containing proteins have been shown to organize protein complexes, while some bind RNA (47,48). Moreover, HEAT domain containing proteins have structural plasticity that allows them to bind to multiple interaction partners, and undergo cargo-induced conformation changes (49,50). For example, ARM repeat containing protein β-catenin is an important organizer for the wnt pathway through its ability to bind multiple proteins at distinct times during the pathway (51). HEAT repeat-containing splicing factor SF3b155 is thought to permit conformational rearrangements of the SF3b complex during the splicing cycle (52). The reported mechanistic functions of ARM/HEAT repeat containing proteins support our proposed protein complex interactions that were not identified by cryo-EM and whose structures may appear to clash with published cryo-EM RESC structures (*i.e*. the GRBC/RESC8 small complex and I-RESC) (21). Cryo-EM provides informative snapshots of distinct RESC complexes; however, it does not inform the nature of transitions such as how GRBC (RESC-A) becomes EC-RESC (RESC-B). The structural plasticity of ARM/HEAT repeat protein, RESC8, likely plays a role in rearranging the spatial conformations of RESC factors, allowing for alternative interaction profiles not observed by cryo-EM. The known functions of ARM/HEAT repeats are consistent with a role for RESC8 in facilitating complex rearrangements leading to the stable interaction between GRBC/gRNA and REMC/RNA. Dynamic complex assembly is also advantageous in that it can allow regulation by intrinsic or extrinsic factors (44). Posttranslational modifications of RESC8 and/or RESC14 could impact the timing of RESC assembly and allow regulatory factors or other complexes such as those involved in RNA degradation to associate. Interestingly, RESC8 is reportedly arginine methylated (53), although the role of this modification in editing awaits study.

Overall, our data are consistent with a model in which RESC14 and RESC8 are critical to the formation and stability of the editing-competent RESC form, allowing for proper RECC association and editing progression. Numerous questions remain, for example, the specific roles of those potential REMC proteins not examined here (RESC7, RESC9), as well as the composition of REMC modules on the pre-edited mRNA. We previously showed that some REMC proteins vary greatly in their abundances, suggesting that REMC modules may not all be equivalent (11). Moreover, the crystal structure of RESC13 indicates that this protein forms a dimer (54) which, although not evident in cryo-EM RESC structures (21), could be the form of the protein on pre-edited mRNA prior to GRBC/REMC association. Finally, we identified by TurboID three hypothetical proteins associating with RESC6 and RESC8 *in vivo*, and whose association with RESC8 is RESC14-dependent. Future studies on the roles of these proteins in U-indel editing or mitochondrial RNA processing will be of great interest.

## Supplementary Material

gkae561_Supplemental_Files

## Data Availability

RNAseq data are available at the Sequence Read Archive. The new sequencing data for A6 and COIII, RESC8 RNAi samples has been deposited under accession number PRJNA986128. Previously published sequencing data is under PRJNA862535 for the A6 and COIII, RESC13 and RESC14 RNAi samples, PRJNA597932 for the A6 and COIII PF 29–13 samples, PRJNA431762 for the RPS12 RESC8 RNAi samples, PRJNA390283 for the RPS12 RESC14 RNAi samples, and PRJNA363102 for the RPS12 RESC13 RNAi and RPS12 control samples. Mass spectrometry data have been deposited to the ProteomeXchange Consortium via the PRIDE partner repository with the dataset identifier PXD046097.

## References

[1] Jensen R.E. , Englund P.T. Network news: the replication of kinetoplast DNA. Annu. Rev. Microbiol. 2012; 66:473–491. 22994497 10.1146/annurev-micro-092611-150057

[2] Aphasizheva I. , Alfonzo J., Carnes J., Cestari I., Cruz-Reyes J., Goringer H.U., Hajduk S., Lukes J., Madison-Antenucci S., Maslov D.A. et al. Lexis and grammar of mitochondrial RNA processing in trypanosomes. Trends Parasitol. 2020; 36:337–355. 32191849 10.1016/j.pt.2020.01.006 PMC7083771

[3] Cruz-Reyes J. , Mooers B.H.M., Doharey P.K., Meehan J., Gulati S. Dynamic RNA holo-editosomes with subcomplex variants: insights into the control of trypanosome editing. Wiley Interdiscipl. Rev. RNA. 2018; 9:e1502. 10.1002/wrna.1502 PMC6185801 30101566

[4] Tarun S.Z. Jr. , Schnaufer A., Ernst N.L., Proff R., Deng J., Hol W., Stuart K KREPA6 is an RNA-binding protein essential for editosome integrity and survival of *Trypanosoma brucei*. RNA. 2008; 14:347–358. 18065716 10.1261/rna.763308 PMC2212256

[5] Schnaufer A. , Panigrahi A.K., Panicucci B., Igo R.P., Wirtz E., Salavati R., Stuart K An RNA ligase essential for RNA editing and survival of the bloodstream form of *Trypanosoma brucei*. Science. 2001; 291:2159–2162. 11251122 10.1126/science.1058955

[6] Koslowsky D. , Sun Y., Hindenach J., Theisen T., Lucas J. The insect-phase gRNA transcriptome in *Trypanosoma brucei*. Nucleic Acids Res. 2014; 42:1873–1886. 24174546 10.1093/nar/gkt973 PMC3919587

[7] Blum B. , Bakalara N., Simpson L. A model for RNA editing in kinetoplastid mitochondria: “guide” RNA molecules transcribed from maxicircle DNA provide the edited information. Cell. 1990; 60:189–198. 1688737 10.1016/0092-8674(90)90735-w

[8] Seiwert S.D. , Stuart K. RNA editing: transfer of genetic information from gRNA to precursor mRNA in vitro. Science. 1994; 266:114–117. 7524149 10.1126/science.7524149

[9] Maslov D.A. , Simpson L. The polarity of editing within a multiple gRNA-mediated domain is due to formation of anchors for upstream gRNAs by downstream editing. Cell. 1992; 70:459–467. 1379519 10.1016/0092-8674(92)90170-h

[10] Koslowsky D.J. , Bhat G.J., Read L.K., Stuart K. Cycles of progressive realignment of gRNA with mRNA in RNA editing. Cell. 1991; 67:537–546. 1718605 10.1016/0092-8674(91)90528-7

[11] Simpson R.M. , Bruno A.E., Chen R., Lott K., Tylec B.L., Bard J.E., Sun Y., Buck M.J., Read L.K. Trypanosome RNA editing mediator complex proteins have distinct functions in gRNA utilization. Nucleic Acids Res. 2017; 45:7965–7983. 28535252 10.1093/nar/gkx458 PMC5737529

[12] Zimmer S.L. , Simpson R.M., Read L.K. High throughput sequencing revolution reveals conserved fundamentals of U-indel editing. Wiley Interdiscipl. Rev. RNA. 2018; 2018:e1487. 10.1002/wrna.1487 PMC6289883 29888550

[13] Gerasimov E.S. , Gasparyan A.A., Afonin D.A., Zimmer S.L., Kraeva N., Lukes J., Yurchenko V., Kolesnikov A. Complete minicircle genome of Leptomonas pyrrhocoris reveals sources of its non-canonical mitochondrial RNA editing events. Nucleic Acids Res. 2021; 49:3354–3370. 33660779 10.1093/nar/gkab114 PMC8034629

[14] Rusche L.N. , Cruz-Reyes J., Piller K.J., Sollner-Webb B. Purification of a functional enzymatic editing complex from *Trypanosoma brucei* mitochondria. EMBO J. 1997; 16:4069–4081. 9233816 10.1093/emboj/16.13.4069 PMC1170030

[15] Aphasizheva I. , Zhang L., Wang X., Kaake R.M., Huang L., Monti S., Aphasizhev R. RNA binding and core complexes constitute the U-insertion/deletion editosome. Mol. Cell. Biol. 2014; 34:4329–4342. 25225332 10.1128/MCB.01075-14 PMC4248751

[16] Kumar V. , Madina B.R., Gulati S., Vashisht A.A., Kanyumbu C., Pieters B., Shakir A., Wohlschlegel J.A., Read L.K., Mooers B.H. et al. REH2C Helicase and GRBC subcomplexes may base pair through mRNA and small guide RNA in kinetoplastid editosomes. J. Biol. Chem. 2016; 291:5753–5764. 26769962 10.1074/jbc.M115.708164 PMC4786712

[17] Madina B.R. , Kumar V., Metz R., Mooers B.H., Bundschuh R., Cruz-Reyes J. Native mitochondrial RNA-binding complexes in kinetoplastid RNA editing differ in guide RNA composition. RNA. 2014; 20:1142–1152. 24865612 10.1261/rna.044495.114 PMC4114691

[18] Kumar V. , Doharey P.K., Gulati S., Meehan J., Martinez M.G., Hughes K., Mooers B.H.M., Cruz-Reyes J. Protein features for assembly of the RNA editing helicase 2 subcomplex (REH2C) in trypanosome holo-editosomes. PLoS One. 2019; 14:e0211525. 31034523 10.1371/journal.pone.0211525 PMC6488192

[19] Kumar V. , Ivens A., Goodall Z., Meehan J., Doharey P.K., Hillhouse A., Hurtado D.O., Cai J.J., Zhang X., Schnaufer A. et al. Site-specific and substrate-specific control of accurate mRNA editing by a helicase complex in trypanosomes. RNA. 2020; 26:1862–1881. 32873716 10.1261/rna.076513.120 PMC7668249

[20] Dubey A.P. , Tylec B.L., Mishra A., Sortino K., Chen R., Sun Y., Read L.K. KREH1 RNA helicase activity promotes utilization of initiator gRNAs across multiple mRNAs in trypanosome RNA editing. Nucleic Acids Res. 2023; 51:5791–5809. 37140035 10.1093/nar/gkad292 PMC10287954

[21] Liu S. , Wang H., Li X., Zhang F., Lee J.K.J., Li Z., Yu C., Hu J.J., Zhao X., Suematsu T. et al. Structural basis of gRNA stabilization and mRNA recognition in trypanosomal RNA editing. Science. 2023; 381:eadg4725. 37410820 10.1126/science.adg4725 PMC10704856

[22] Ammerman M.L. , Downey K.M., Hashimi H., Fisk J.C., Tomasello D.L., Faktorova D., Kafkova L., King T., Lukes J., Read L.K. Architecture of the trypanosome RNA editing accessory complex, MRB1. Nucleic Acids Res. 2012; 40:5637–5650. 22396527 10.1093/nar/gks211 PMC3384329

[23] Dubey A.P. , Tylec B.L., McAdams N.M., Sortino K., Read L.K. Trypanosome RNA Editing Substrate binding Complex integrity and function depends on the upstream action of RESC10. Nucleic Acids Res. 2021; 49:3557–3572. 33677542 10.1093/nar/gkab129 PMC8034615

[24] McAdams N.M. , Harrison G.L., Tylec B.L., Ammerman M.L., Chen R., Sun Y., Read L.K. MRB10130 is a RESC assembly factor that promotes kinetoplastid RNA editing initiation and progression. RNA. 2019; 25:1177–1191. 31221726 10.1261/rna.071902.119 PMC6800514

[25] McAdams N.M. , Simpson R.M., Chen R., Sun Y., Read L.K. MRB7260 is essential for productive protein–RNA interactions within the RNA editing substrate binding complex during trypanosome RNA editing. RNA. 2018; 24:540–556. 29330168 10.1261/rna.065169.117 PMC5855954

[26] Wirtz E. , Leal S., Ochatt C., Cross G.A. A tightly regulated inducible expression system for conditional gene knock-outs and dominant-negative genetics in *Trypanosoma brucei*. Mol. Biochem. Parasitol. 1999; 99:89–101. 10215027 10.1016/s0166-6851(99)00002-x

[27] Pelletier M. , Read L.K. RBP16 is a multifunctional gene regulatory protein involved in editing and stabilization of specific mitochondrial mRNAs in *Trypanosoma brucei*. RNA. 2003; 9:457–468. 12649497 10.1261/rna.2160803 PMC1370412

[28] Dean S. , Sunter J., Wheeler R.J., Hodkinson I., Gluenz E., Gull K. A toolkit enabling efficient, scalable and reproducible gene tagging in trypanosomatids. Open Biology. 2015; 5:140197. 25567099 10.1098/rsob.140197 PMC4313374

[29] Schimanski B. , Nguyen T.N., Gunzl A. Highly efficient tandem affinity purification of trypanosome protein complexes based on a novel epitope combination. Eukaryot. Cell. 2005; 4:1942–1950. 16278461 10.1128/EC.4.11.1942-1950.2005 PMC1287860

[30] Silverman J.S. , Schwartz K.J., Hajduk S.L., Bangs J.D. Late endosomal Rab7 regulates lysosomal trafficking of endocytic but not biosynthetic cargo in *Trypanosoma brucei*. Mol. Microbiol. 2011; 82:664–678. 21923766 10.1111/j.1365-2958.2011.07842.x PMC4324464

[31] Smith J.T. Jr. , Dolezelova E., Tylec B., Bard J.E., Chen R., Sun Y., Zikova A., Read L.K Developmental regulation of edited CYb and COIII mitochondrial mRNAs is achieved by distinct mechanisms in *Trypanosoma brucei*. Nucleic Acids Res. 2020; 48:8704–8723. 32738044 10.1093/nar/gkaa641 PMC7470970

[32] Sortino K. , Tylec B.L., Chen R., Sun Y., Read L.K. Conserved and transcript-specific functions of the RESC factors, RESC13 and RESC14, in kinetoplastid RNA editing. RNA. 2022; 28:1496–1508. 36096641 10.1261/rna.079389.122 PMC9745829

[33] Simpson R.M. , Bruno A.E., Bard J.E., Buck M.J., Read L.K. High-throughput sequencing of partially edited trypanosome mRNAs reveals barriers to editing progression and evidence for alternative editing. RNA. 2016; 22:677–695. 26908922 10.1261/rna.055160.115 PMC4836643

[34] Subrtova K. , Panicucci B., Zikova A. ATPaseTb2, a unique membrane-bound FoF1-ATPase component, is essential in bloodstream and dyskinetoplastic trypanosomes. PLoS Pathog. 2015; 11:e1004660. 25714685 10.1371/journal.ppat.1004660 PMC4340940

[35] Hierro-Yap C. , Subrtova K., Gahura O., Panicucci B., Dewar C., Chinopoulos C., Schnaufer A., Zikova A. Bioenergetic consequences of F(o)F(1)-ATP synthase/ATPase deficiency in two life cycle stages of *Trypanosoma brucei*. J. Biol. Chem. 2021; 296:100357. 33539923 10.1016/j.jbc.2021.100357 PMC7949148

[36] Pyrih J. , Raskova V., Skodova-Sverakova I., Panek T., Lukes J. ZapE/Afg1 interacts with Oxa1 and its depletion causes a multifaceted phenotype. PLoS One. 2020; 15:e0234918. 32579605 10.1371/journal.pone.0234918 PMC7314023

[37] Kim D.I. , Jensen S.C., Roux K.J. Identifying protein–protein associations at the nuclear envelope with BioID. Methods Mol. Biol. 2016; 1411:133–146. 27147039 10.1007/978-1-4939-3530-7_8 PMC5473025

[38] Shen S. , An B., Wang X., Hilchey S.P., Li J., Cao J., Tian Y., Hu C., Jin L., Ng A. et al. Surfactant cocktail-aided extraction/precipitation/on-pellet digestion strategy enables efficient and reproducible sample preparation for large-scale quantitative proteomics. Anal. Chem. 2018; 90:10350–10359. 30078316 10.1021/acs.analchem.8b02172

[39] Shen S. , Wang X., Zhu X., Rasam S., Ma M., Huo S., Qian S., Zhang M., Qu M., Hu C. et al. High-quality and robust protein quantification in large clinical/pharmaceutical cohorts with IonStar proteomics investigation. Nat. Protoc. 2023; 18:700–731. 36494494 10.1038/s41596-022-00780-w PMC10673696

[40] Ammerman M.L. , Hashimi H., Novotna L., Cicova Z., McEvoy S.M., Lukes J., Read L.K. MRB3010 is a core component of the MRB1 complex that facilitates an early step of the kinetoplastid RNA editing process. RNA. 2011; 17:865–877. 21451155 10.1261/rna.2446311 PMC3078736

[41] Davidge B. , McDermott S.M., Carnes J., Lewis I., Tracy M., Stuart K.D. Multiple domains of the integral KREPA3 protein are critical for the structure and precise functions of RNA editing catalytic complexes in *Trypanosoma brucei*. RNA. 2023; 29:1591–1609. 37474258 10.1261/rna.079691.123 PMC10578492

[42] Weng J. , Aphasizheva I., Etheridge R.D., Huang L., Wang X., Falick A.M., Aphasizhev R. Guide RNA-binding complex from mitochondria of trypanosomatids. Mol. Cell. 2008; 32:198–209. 18951088 10.1016/j.molcel.2008.08.023 PMC2645705

[43] Hashimi H. , Cicova Z., Novotna L., Wen Y.Z., Lukes J. Kinetoplastid guide RNA biogenesis is dependent on subunits of the mitochondrial RNA binding complex 1 and mitochondrial RNA polymerase. RNA. 2009; 15:588–599. 19228586 10.1261/rna.1411809 PMC2661843

[44] Beusch I. , Madhani H.D. Understanding the dynamic design of the spliceosome. Trends Biochem. Sci. 2024; 10.1016/j.tibs.2024.03.012. 38641465

[45] Kirby L.E. , Koslowsky D Cell-line specific RNA editing patterns in *Trypanosoma brucei* suggest a unique mechanism to generate protein variation in a system intolerant to genetic mutations. Nucleic Acids Res. 2020; 48:1479–1493. 31840176 10.1093/nar/gkz1131 PMC7026638

[46] Meehan J. , McDermott S.M., Ivens A., Goodall Z., Chen Z., Yu Z., Woo J., Rodshagen T., McCleskey L., Sechrist R. et al. Trypanosome RNA helicase KREH2 differentially controls non-canonical editing and putative repressive structure via a novel proposed ‘bifunctional’ gRNA in mRNA A6. Nucleic Acids Res. 2023; 51:6944–6965. 37246647 10.1093/nar/gkad453 PMC10359474

[47] Kim I. , Kwak H., Lee H.K., Hyun S., Jeong S. beta-catenin recognizes a specific RNA motif in the cyclooxygenase-2 mRNA 3'-UTR and interacts with HuR in colon cancer cells. Nucleic Acids Res. 2012; 40:6863–6872. 22544606 10.1093/nar/gks331 PMC3413138

[48] Stein A.J. , Fuchs G., Fu C., Wolin S.L., Reinisch K.M. Structural insights into RNA quality control: the ro autoantigen binds misfolded RNAs via its central cavity. Cell. 2005; 121:529–539. 15907467 10.1016/j.cell.2005.03.009 PMC1769319

[49] Friedrich D. , Marintchev A., Arthanari H. The metaphorical swiss army knife: the multitude and diverse roles of HEAT domains in eukaryotic translation initiation. Nucleic Acids Res. 2022; 50:5424–5442. 35552740 10.1093/nar/gkac342 PMC9177959

[50] Yoshimura S.H. , Hirano T. HEAT repeats - versatile arrays of amphiphilic helices working in crowded environments?. J. Cell Sci. 2016; 129:3963–3970. 27802131 10.1242/jcs.185710

[51] Xu W. , Kimelman D Mechanistic insights from structural studies of beta-catenin and its binding partners. J. Cell Sci. 2007; 120:3337–3344. 17881495 10.1242/jcs.013771

[52] Cretu C. , Schmitzova J., Ponce-Salvatierra A., Dybkov O., De Laurentiis E.I., Sharma K., Will C.L., Urlaub H., Luhrmann R., Pena V. Molecular architecture of SF3b and structural consequences of its cancer-related mutations. Mol. Cell. 2016; 64:307–319. 27720643 10.1016/j.molcel.2016.08.036

[53] Fisk J.C. , Li J., Wang H., Aletta J.M., Qu J., Read L.K. Proteomic analysis reveals diverse classes of arginine methylproteins in mitochondria of trypanosomes. Mol. Cell. Proteomics. 2013; 12:302–311. 23152538 10.1074/mcp.M112.022533 PMC3567855

[54] Travis B. , Shaw P.L.R., Liu B., Ravindra K., Iliff H., Al-Hashimi H.M., Schumacher M.A. The RRM of the kRNA-editing protein TbRGG2 uses multiple surfaces to bind and remodel RNA. Nucleic Acids Res. 2019; 47:2130–2142. 30544166 10.1093/nar/gky1259 PMC6393287

