# Supplementary material for: RESC14 and RESC8 cooperate to mediate RESC function and dynamics during trypanosome RNA editing
Source: Nucleic Acids Res. 2024 Jul 5;52(16):9867–85. doi: 10.1093/nar/gkae561 (PMC11381364; doi:10.1093/nar/gkae561)
Supplement: gkae561_Supplemental_Files [file gkae561_supplemental_files.zip › Supplemental_Figs_S1_to_S5.pdf]

Supplementary Materials for

RESC14 and RESC8 cooperate to mediate RESC function and dynamics during trypanosome RNA editing

Katherine Sortino[1], Xiaoyu Zhu[2], Shichen Shen[2], Ming Zhang[2], Jun Qu[2], and Laurie K. Read[1,3]

[1]Department of Microbiology, Jacobs School of Medicine and Biomedical Sciences;
[2]Department of Pharmaceutical Sciences, University at Buffalo and NYS Center of Excellence in Bioinformatics and Life Sciences, University at Buffalo, USA

The file includes: Figs. S1 to S5 with legends

Other Supplementary Material for this manuscript includes the following: Tables S1 to S8

[3]To whom correspondence should be addressed:
Email: lread@buffalo.edu
Telephone: 716-829-3307

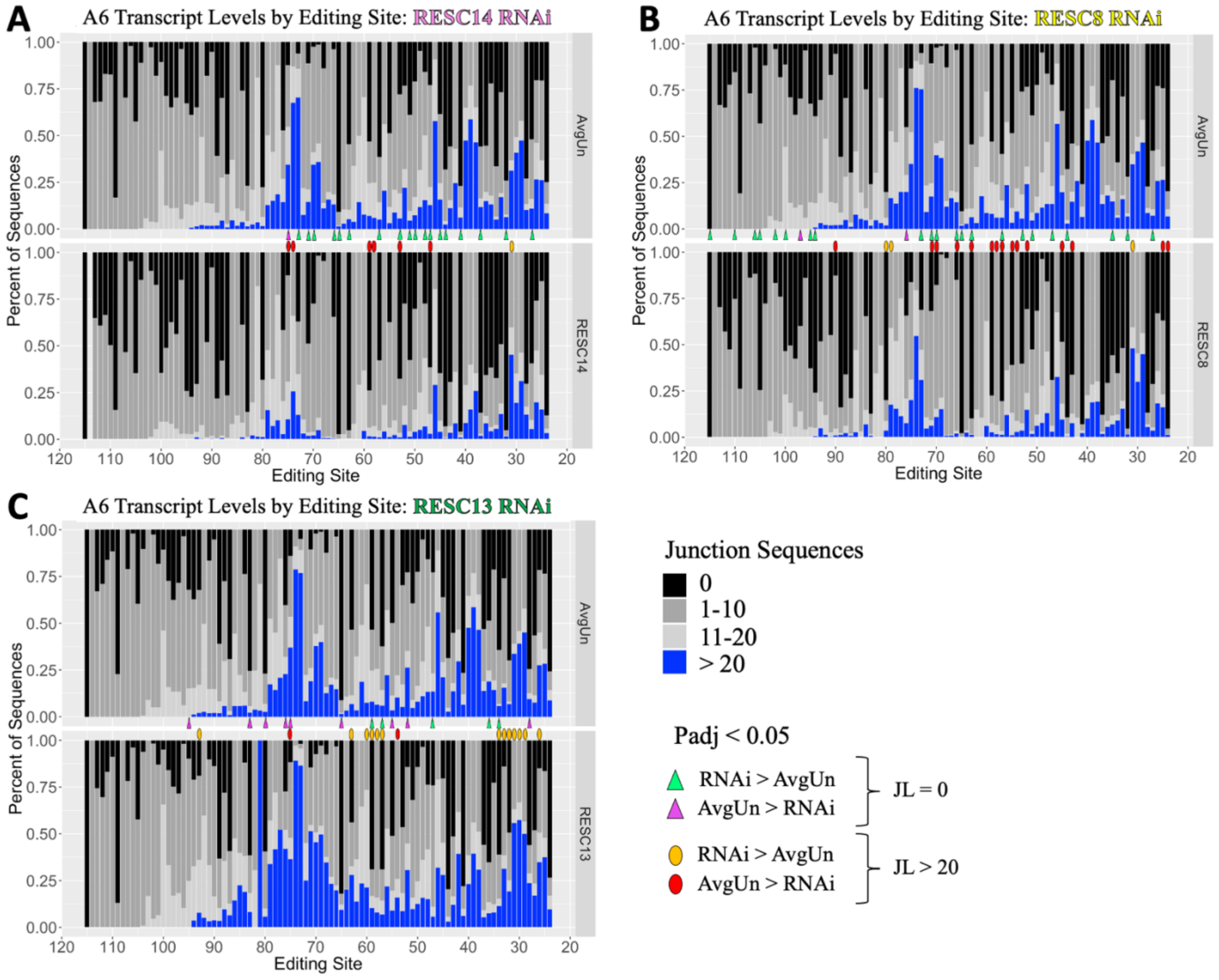
A
A6 Transcript Levels by Editing Site: RESC14 RNAi
B
A6 Transcript Levels by Editing Site: RESC8 RNAi
C
A6 Transcript Levels by Editing Site: RESC13 RNAi
Percent of Sequences
Editing Site
AvgUn
RESC14
RESC8
RESC13
Junction Sequences
0
1-10
11-20
> 20
Padj < 0.05
RNAi > AvgUn
AvgUn > RNAi
JL = 0
RNAi > AvgUn
AvgUn > RNAi
JL > 20

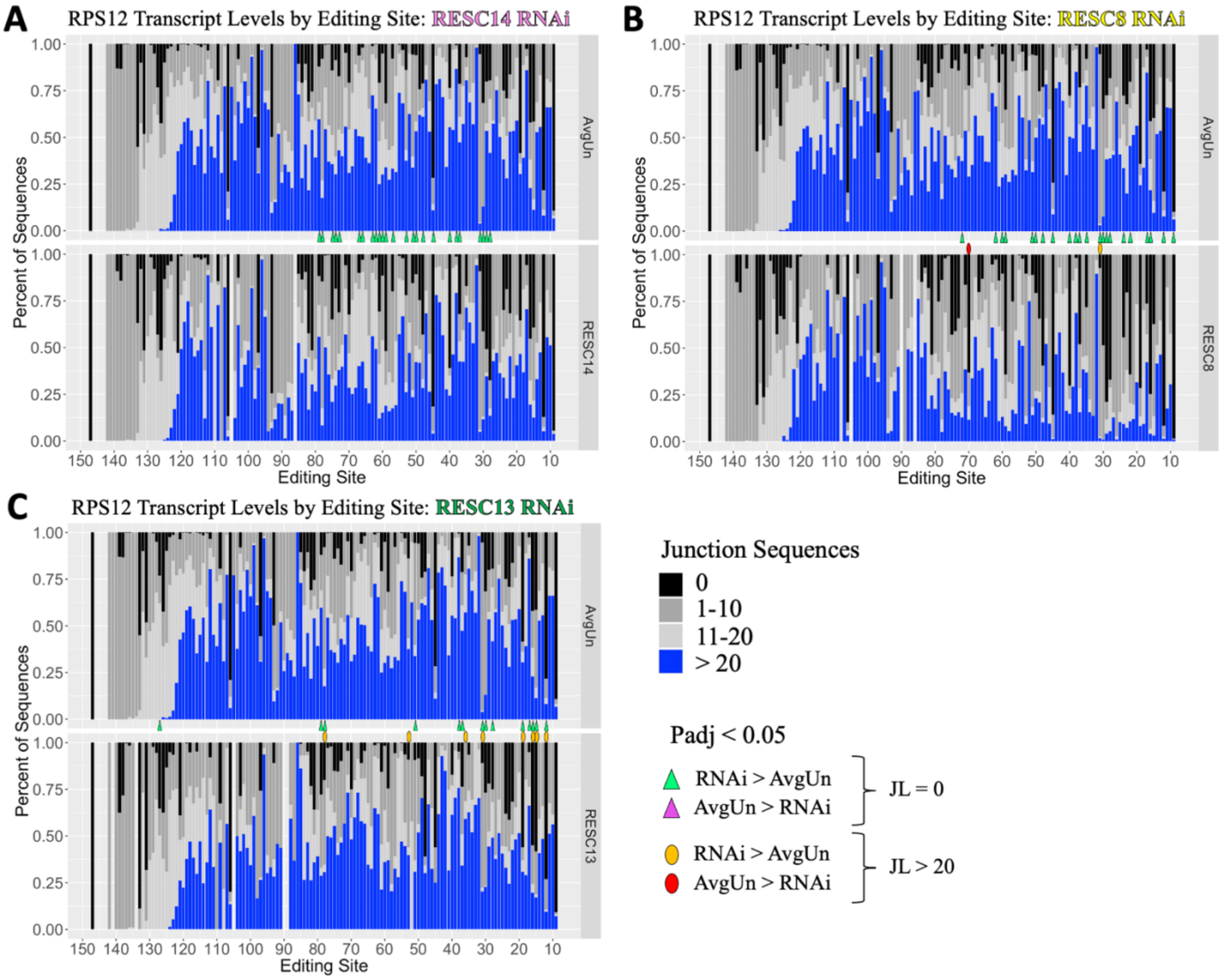

Supplemental Fig. 1 and 2. Changes in junction lengths upon RESC14, RESC8, or RESC13 depletion. The percent of sequences with junction lengths (JL) of 0 (black bars), 1-10 (light grey bars), 11-20 (dark grey bars), and greater than 20 (blue bars) at each editing site across A6 mRNA (**Fig. S1**) and RPS12 mRNA (**Fig. S2**) for RESC14 (**A**), RESC8 (**B**) and RESC13 (**C**) RNAi cells, compared to control samples. Symbols as in **Fig. 2**.

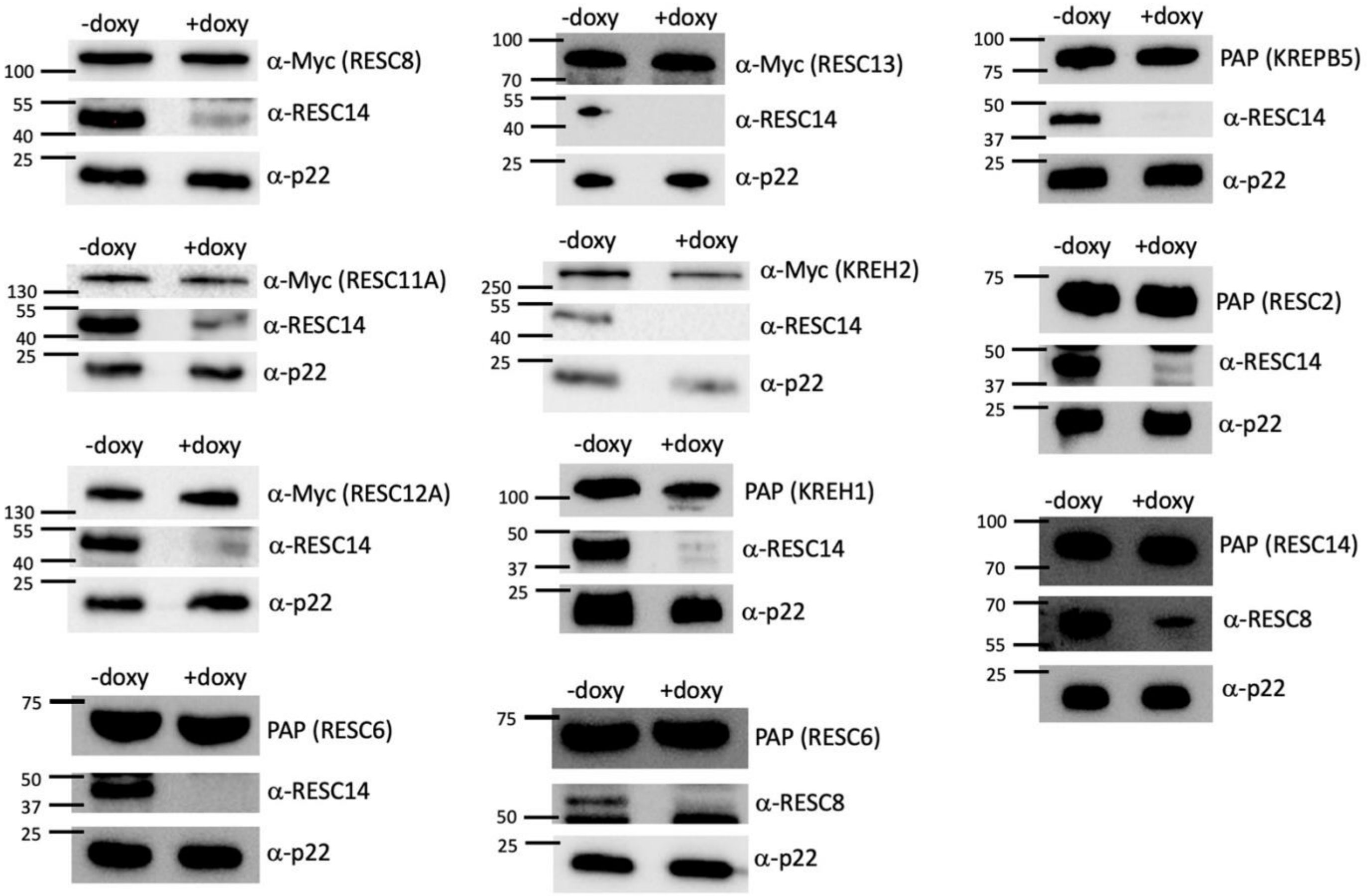

Supplemental Fig. 3: Validation of cell lines used in blue native PAGE analysis. Tagged cell lines were induced 3 days for RESC14 RNAi or 2 days for RESC8 RNAi. Cells were harvested and analyzed by western blot using antibodies against the tag of tagged proteins and specific antibodies to the protein knocked down. Anti-myc detects the MHT tag of RESC12A, RESC11A, RESC13, RESC8, and KREH2. PAP recognizes the Protein A region of the MHT tag for KREPB5, KREH1 and RESC14, as well as the PTP tag of RESC2 and RESC6. p22 is the loading control.

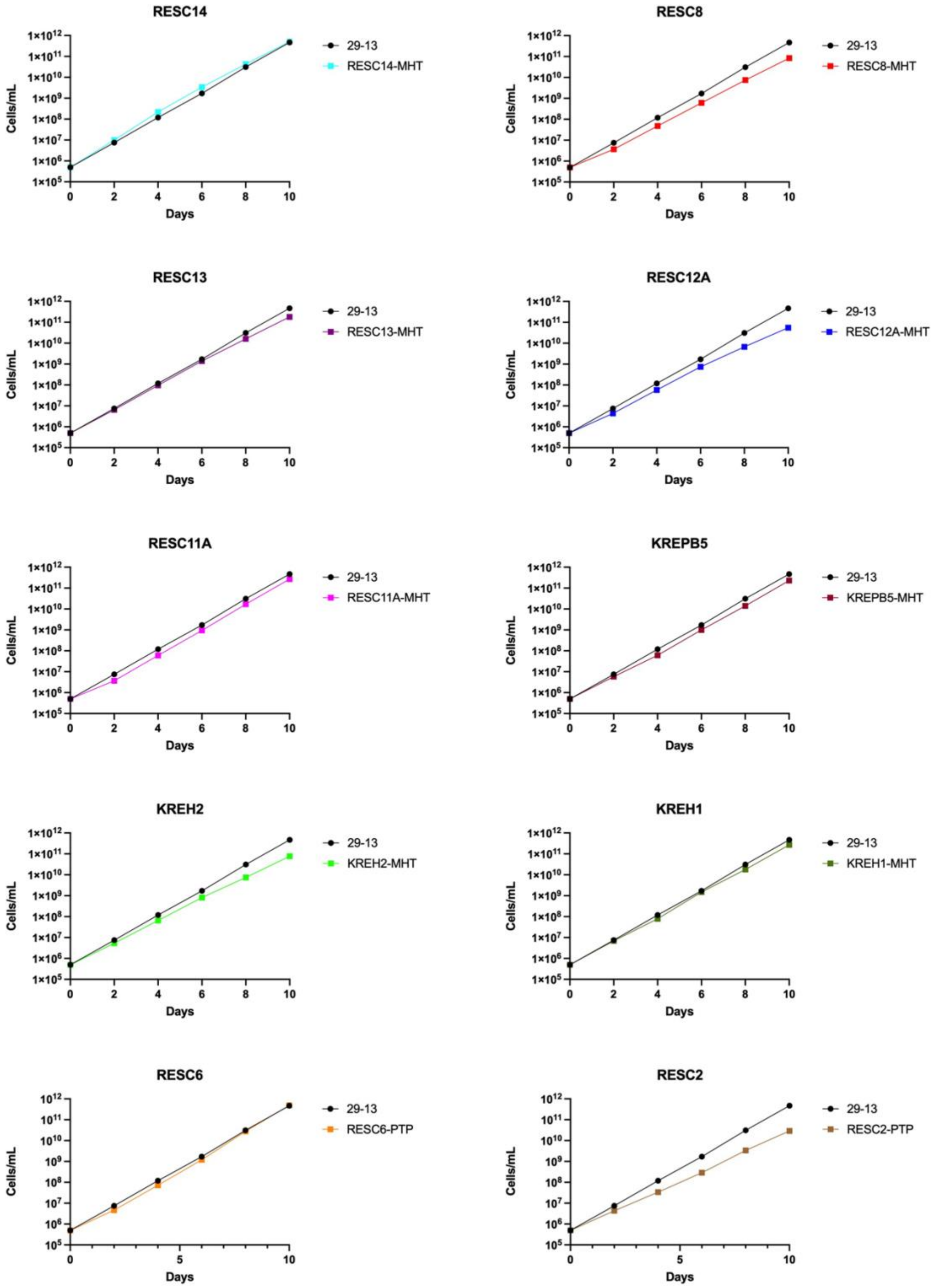

Supplemental Fig. 4: Growth of cell lines used in blue native PAGE analysis. Cell growth was measured for one replicate each of MHT-tagged RESC14, RESC8, RESC13, RESC12A, RESC11A, KREH2, KREH1 and KREPB5, and PTP-tagged RESC6 and RESC2 in the RESC14 RNAi background. The growth of these tagged cell lines was compared to the growth of wild-type 29-13 cells. There is no substantial growth defect for any of the tagged cell lines.

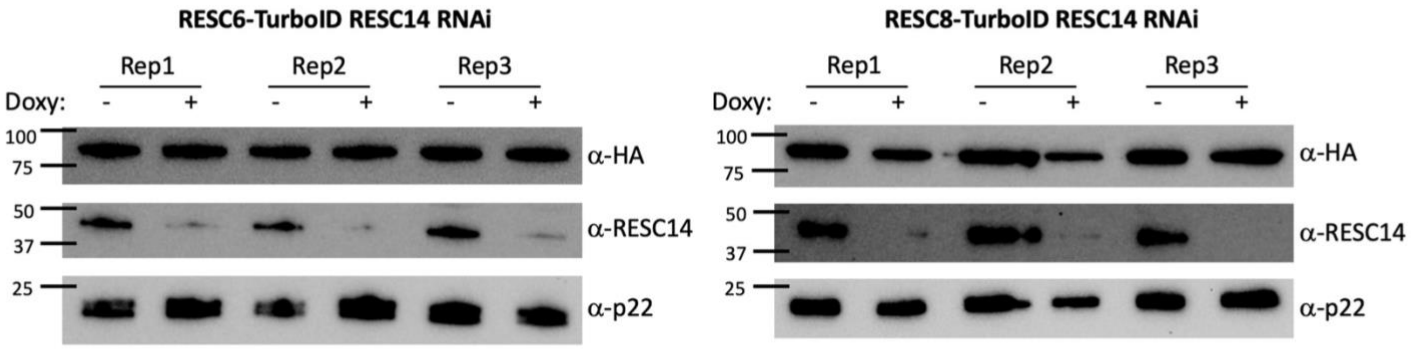

Supplemental Fig. 5: Validation of cell lines used for TurboID. Western blot analysis of cell lysates from the three replicates of RESC6-TurboID and RESC8-TurboID cells that were sent for mass spectrometry analysis, to confirm the presence of the TurboID tag and successful RESC14 RNAi. RESC14 RNAi was induced for 3 days and anti-RESC14 was used to check the level of knockdown. We blotted with anti-HA to check the TurboID tag, and anti-p22 was used as the loading control.
